# Full-time sequence assessment of okra seedling vigor under salt stress based on leaf area and leaf growth rate estimation using the YOLOv11-HSECal instance segmentation model

**DOI:** 10.3389/fpls.2025.1625154

**Published:** 2025-08-14

**Authors:** Xiaowei Cao, Yifan Li, Yaben Zhang, Zhibo Zhong, Ruxiao Bai, Peng Yang, Feng Pan, Xiuqing Fu

**Affiliations:** ^1^ College of Engineering, Nanjing Agricultural University, Nanjing, China; ^2^ Institute of Farmland Water Conservancy and Soil-Fertilizer, Xinjiang Academy of Agricultural Reclamation Science, Shihezi, Xinjiang, China; ^3^ Institute of Mechanical Equipment, Xinjiang Academy of Agricultural Reclamation Science, Shihezi, Xinjiang, China

**Keywords:** YOLOv11-HSECal model, okra, salt stress, time-series, leaf area, leaf growth rate, plant vitality evaluation

## Abstract

**Introduction:**

With the growing severity of global salinization, assessing plant growth vitality under salt stress has become a critical aspect in agricultural research.

**Methods:**

In this paper, a method for calculating the leaf area and leaf growth rate of okra based on the YOLOv11-HSECal model is proposed, which is used to evaluate the activity of okra at the seedling stage. A high-throughput, Full-Time Sequence Crop Germination Vigor Monitoring System was developed to automatically capture image data from seed germination to seedling growth stage, while maintaining stable temperature and lighting conditions. To address the limitations of the traditional YOLOv11-seg model, the YOLOv11-HSECal model was optimized by incorporating the HGNetv2 backbone, Slim-Neck feature fusion, and EMAttention mechanisms.

**Results:**

These improvements led to a 1.1% increase in mAP50, a 0.6% reduction in FLOPs, and a 14.1% decrease in model parameters. Additionally, Merge and Cal modules were integrated for calculating the leaf area and growth rate of okra seedlings. Finally, through salt stress experiments, we assessed the effects of varying NaCl concentrations (CK, 10 mmol/L, 20 mmol/L, 30 mmol/L, 40 mmol/L, 50 mmol/L, and 60 mmol/L) on the leaf area and growth rate of okra seedlings, verifying the inhibitory effects of salt stress on seedling vitality.

**Discussion:**

The results demonstrate that the YOLOv11-HSECal model efficiently and accurately evaluates okra seedling growth vitality under salt stress in a full-time monitoring manner, offering significant potential for broader applications. This work provides a novel solution for full-time plant growth monitoring and vitality assessment in smart agriculture and offers valuable insights into the impact of salt stress on crop growth.

## 1 Introduction

With the rapid increase in the global population, the production of vegetables and grains has become a primary concern (Zhu et al., 2023). The vitality of plant seedlings is a key determinant of the speed and uniformity of plant emergence and early growth, directly influencing their ability to acquire and compete for essential resources (Mondo et al., 2013). The yield of crops is closely linked to seedling vitality: stronger vitality at the seedling stage correlates with higher yields under identical growth conditions (Podlaski and Chomontowski, 2020). Plants with higher vitality during the seedling stage exhibit better performance in resource acquisition (such as light, water, and nutrients) and enhanced competitiveness, particularly in competition with weeds. Additionally, high-vitality seeds tend to have larger leaf areas during the seedling stage compared to those of low-vitality seeds. For example, during the four-leaf and eight-leaf stages of maize, the leaf area index of high-vitality seeds is 37% and 16% greater than that of low-vitality seeds, respectively (Mondo et al., 2013). Assessing seedling vitality through leaf area and growth rate during this stage is an essential method for plant selection and breeding. Okra (Abelmoschus esculentus L.), an annual herbaceous plant belonging to the Malvaceae family, is a specialty vegetable with both high nutritional and economic value. Its tender pods are rich in proteins, dietary fiber, minerals, and various vitamins, with protein content surpassing that of most conventional fruits and vegetables, and offering a well-balanced amino acid profile. As an economic crop integrating edible, medicinal, and industrial functions, okra is characterized by a short cultivation cycle, strong environmental adaptability, and high yield per unit area. In addition to fresh consumption, it is extensively used in the production of value-added products such as frozen food, seed oil, and health supplements. Due to these advantages, okra has been widely cultivated across the globe (Elkhalifa et al., 2021). Salt stress is a major challenge in global agriculture, with approximately 21% of arable land in China affected by salinization (Wang et al., 2013). Soil salinization has emerged as one of the principal environmental stressors limiting plant productivity (Yu et al., 2019; Wang L. et al., 2022). Salt stress, particularly during the seedling stage, significantly reduces photosynthetic efficiency, impairs plant biomass accumulation, and inhibits leaf expansion (Zhang et al., 2018; Zhao et al., 2022). Studies have shown that salt stress leads to a reduction in leaf area and chlorophyll content in okra, causing overall growth retardation and potentially inducing oxidative stress (Abbas et al., 2017). Therefore, accurately assessing the leaf area and growth rate of okra seedlings under salt stress is essential for improving seed quality, enhancing yield, and achieving sustainable agricultural objectives.

Traditional methods for measuring leaf area primarily rely on manual techniques, such as the cardboard drawing method (Korva and Forbes, 1997), the weighing method (Castillo et al., 2014), volumetric accelerated leaf area measurement (Lee et al., 2022), and laser scanning (Berk et al., 2020). Although these methods provide high accuracy, most are destructive and involve cumbersome, time-consuming operations. To reduce manual intervention and enhance efficiency, researchers have developed various image-based, non-destructive leaf area estimation methods, including modified models based on leaf length and width (Leroy et al., 2007), segmentation algorithms based on color and shape features (Tu et al., 2021), and estimation methods utilizing leaf surface density constants (Tu et al., 2021). Meanwhile, An et al. developed an automated high-throughput phenotyping pipeline that utilizes a cost-effective imaging system combined with image processing algorithms to generate 2D orthomosaic projections (An et al., 2016). While these approaches improve measurement efficiency, they remain (Castillo et al., 2014) sensitive to factors such as background, lighting, and leaf structure, which limits their accuracy. In recent years, advances in 3D reconstruction and imaging technologies have led researchers to explore methods such as binocular stereo vision (Gong et al., 2015), infrared thermal imaging (Zhang and Zhang, 2022), and LIDAR measurement systems (Hu et al., 2018) for leaf area measurement. These techniques offer non-contact, high-precision 3D reconstruction of leaves, addressing some of the limitations associated with occlusion and projection errors inherent in 2D methods. However, their high equipment costs, complex algorithms, challenges in achieving real-time measurements, and sensitivity to environmental changes continue to hinder their widespread adoption in conventional agricultural management (Hu et al., 2018). Therefore, there is an urgent need to develop a novel method for measuring leaf area and leaf growth rate that is high-precision, full-time-series, non-invasive, robust to environmental variations, and non-destructive. Such a method would better support modern crop growth monitoring and meet the practical demands of intelligent agriculture.

In recent years, deep learning-based instance segmentation techniques have made remarkable advances in agricultural image analysis, significantly enhancing the efficiency and accuracy of plant phenotypic feature extraction. Compared to traditional image processing and shallow machine learning methods, deep learning models exhibit superior feature learning capabilities and adaptability across diverse environments. Among them, the YOLO (You Only Look Once) series has been widely adopted in agricultural applications due to its end-to-end architecture and excellent balance between speed and accuracy. For instance, Chen et al. (2025) proposed the GE-YOLO model, which incorporates a Gold YOLO multi-scale fusion structure and an EMA attention mechanism, effectively improving weed detection performance in rice fields. Miao et al. (2025) introduced SerpensGate-YOLOv8, integrating DySnakeConv and STA modules to enhance the model’s perception of curved edges in plant disease regions. Similarly, Lv and Su (2024) developed YOLOv5-CBAM-C3TR by introducing a Transformer-based attention mechanism, significantly boosting inter-category segmentation accuracy for apple leaf disease detection. Li HK. et al. (2024) proposed the RSG-YOLOv8 model, which integrates CSPDenseNet and the BRA module for improved detection of extremely small targets during rice seed germination. Additionally, Zhang et al. (2024) developed the S-T-YOLOv5 model, combining the Swin Transformer architecture to achieve high-precision, highly adaptive pollen counting in multiple plant species such as alfalfa. Jiang et al., 2023 utilized the YOLOv8-Pea network to assess the drought tolerance of pea seeds, while Fu et al., 2022 evaluated salt tolerance during wheat seed germination using a YOLOv4-based model. Moreover, Wang ZP. et al. (2022) and Cui et al., 2023 applied optimized YOLOv5 and YOLOv4-Tiny models, respectively, to apple stem recognition and pinecone harvesting tasks. Despite YOLO’s strong performance in object detection tasks, its bounding-box-based mechanism cannot provide pixel-level contour information, limiting its effectiveness for applications such as precise leaf area estimation, where high boundary accuracy is critical. In natural field environments, plant leaves often exhibit complex structures, including overlap, occlusion, distortion, and non-rigid deformations. Relying solely on bounding box detection makes it challenging to achieve accurate single-leaf segmentation and area calculation. To overcome these limitations, researchers have increasingly adopted instance segmentation approaches for plant leaf recognition and area estimation. For example, Huang et al. (2024) proposed a Mask R-CNN model enhanced with local refinement mechanisms to achieve fine segmentation of Chinese cabbage leaves under complex greenhouse conditions. Lüling et al. (2023) combined Mask R-CNN with Structure-from-Motion (SfM) 3D reconstruction technology to enable non-contact measurement of fruit volume and leaf area across multiple cabbage growth stages. Lu et al. (2024) developed a maize growth organ recognition and annotation system by integrating YOLOv5 with the Segment Anything Model. Furthermore, the Hierarchical Plant Segmentation Framework (Roggiolani et al., 2023) enabled semantic segmentation without relying on point clouds, achieving joint modeling of plant and leaf instances and providing a decoupled multi-scale pathway for leaf area estimation. Schneider et al. (2024) employed YOLOv8 to detect and model different growth stages of chili peppers in hydroponic systems. Although previous studies have demonstrated significant improvements in segmentation accuracy using various YOLO-based models, they still suffer from several limitations, including complex network architectures, lack of inherent segmentation capabilities, large parameter sizes, low inference efficiency, and limited robustness under natural lighting conditions (Khanam and Hussain, 2024). Moreover, most existing approaches primarily focus on static images or a single growth stage, making them unsuitable for high-throughput monitoring of leaf area throughout the entire plant growth cycle.YOLOv11 (Wu et al., 2024), as the latest open-source iteration of the YOLO series, exhibits comprehensive improvements over earlier versions in terms of input image processing, computational load, edge device deployment, precision, and parameter efficiency. It addresses the shortcomings of earlier variants that primarily focused on irregular single-frame images and were constrained by environmental complexity. YOLOv11 is thus better suited for executing full-growth-cycle object detection and instance segmentation tasks with higher efficiency. In addition, conventional YOLO models tend to underperform in recognizing small features and irregular leaf contours. Therefore, further model improvements to enhance segmentation precision are necessary. When targeting only okra leaf detection and segmentation, the output merely provides the number of segmented masks in an image. To address this, the YOLO model needs to be extended with post-processing modules that refine the segmentation output and enable further image-level analysis. Furthermore, the model still exhibits limitations in terms of FLOPs and parameter count, imposing high requirements for computational resources, runtime, and battery life—factors that challenge its deployment on modern agricultural devices. Thus, reducing FLOPs and model parameters is essential to achieve further lightweighting and improve deployment efficiency in resource-constrained agricultural environments.

In response to the aforementioned challenges, this study designs a full-time-series crop germination vigor monitoring system and proposes an improved YOLOv11-HSECal model to achieve the following objectives:

1. Address the limitations of existing methods in complex environments, including insufficient accuracy, destructive data collection, and irregular phenotypic monitoring, by enabling non-contact and dynamic leaf data acquisition throughout the entire growth process of okra from seed germination to the seedling stage.
2. Meet the agricultural monitoring demands for lightweight, high-throughput, and high-precision deep learning algorithms, while ensuring large-scale deployment on resource-constrained devices.
3. Accurately assess and output the actual leaf area and leaf growth rate of okra under salt stress, enabling effective monitoring of okra seedling vigor. This provides a viable solution to the low accuracy of YOLO models in detecting small features and irregular contours, and to their inability to output beyond instance masks.

## 2 Materials and methods

### 2.1 Development of full time sequence monitoring system for plant initial growth

**Figure 1:**
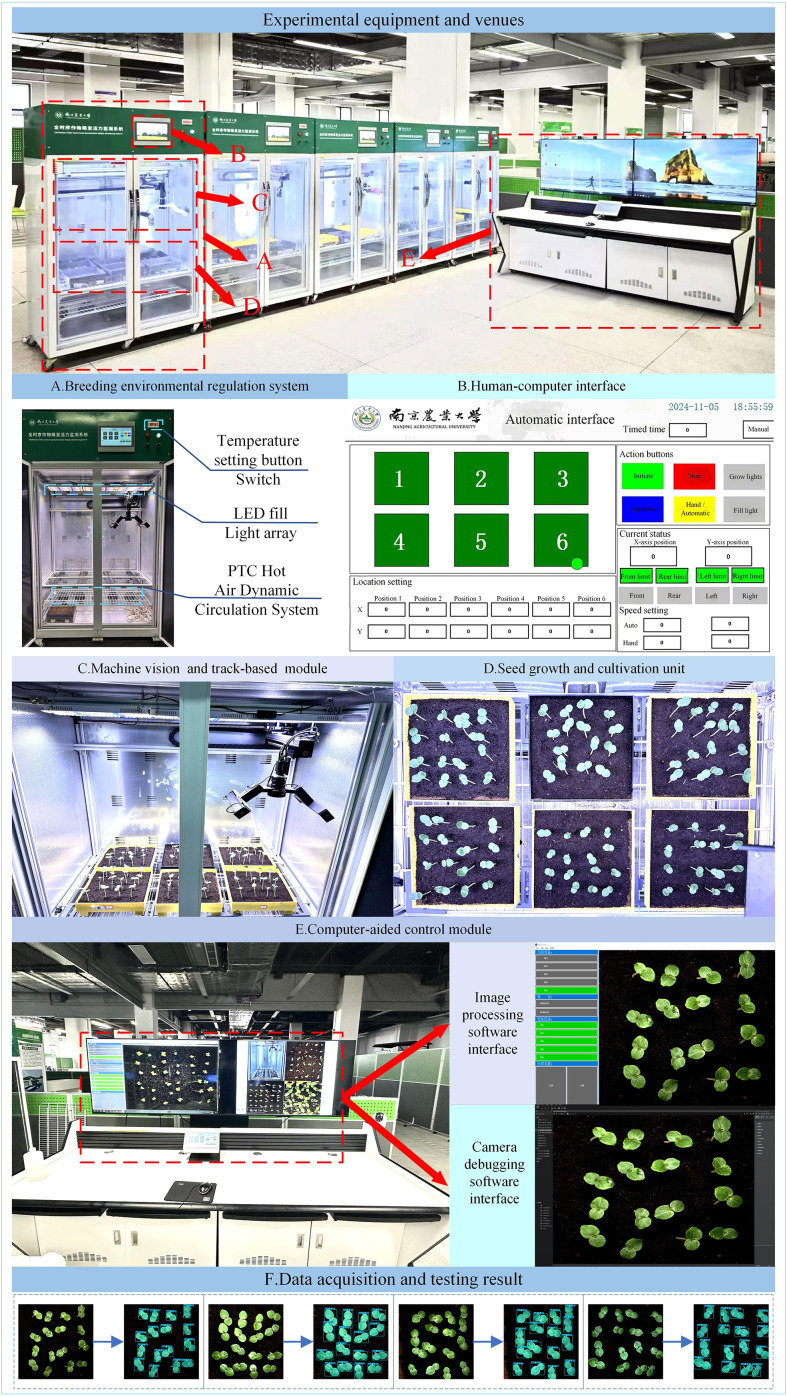
Full-time sequence crop germination vigor monitoring system: **(A)** Breeding environmental regulation system; **(B)** Human-computer interface; **(C)** Machine vision and track-based module; **(D)** Seed growth and cultivation unit; **(E)** Computer-aided control module; **(F)** Data acquisition and testing result.

To enable full-time monitoring of okra seedling growth, we developed a high-throughput, full-time crop germination vigor monitoring system capable of maintaining optimal growth conditions for plants, as illustrated in **Figure 1**. This system supports multiple breeding methods, including soil cultivation and hydroponics, while providing a controlled environment with constant temperature and continuous lighting. Furthermore, it enables the automated and continuous monitoring of the entire developmental process from seed germination to seedling growth, referred to as “ Temporal monitoring”. The system is composed of five major components: (1) breeding environment control system, (2) machine vision-based high-throughput imaging and orbital image acquisition module, (3) seed growth and cultivation unit, (4) computer-aided control module, and (5) human-computer interaction interface.

The breeding environment control system (**Figure 1A**) consists of an incubator measuring 1055 mm in length, 740 mm in width, and 1740 mm in height (manufacturer: Henan Greentech Electric Technology Co., Ltd.), with a total of five incubators deployed at the experimental site. This system primarily controls lighting and temperature. It integrates a dual-channel temperature control system featuring two precision heaters (operating range: 10–60°C; accuracy: ± 0.1°C) equipped with Tp-100 thermocouples and an embedded PTC thermodynamic circulation unit. Together, they form a dynamic temperature control network capable of intelligently adjusting heating power based on real-time data feedback. When the internal temperature falls below the set threshold, the PTC hot aerodynamic circulation system activates to raise the temperature; conversely, when the temperature exceeds the upper limit, the system automatically shuts down to maintain environmental stability. Temperature settings are located at the upper right corner of the incubator and can be specified using the adjustment button. The lighting system features a dual-sided and top-mounted fixed LED array with a non-dimmable intensity design, controlled via a switch on the incubator panel. It provides full-cycle lighting conditions to support the seedling growth of okra.

The machine vision-based high-throughput imaging and track-based image acquisition module (**Figure 1C**) was constructed using a two-dimensional precision motion platform, with an X-axis horizontal rail spacing of 800 mm and a Y-axis vertical rail spacing of 1000 mm. A camera was mounted on a stepper motor-driven rail, allowing free movement across the upper plane of the incubator and enabling image capture at six designated positions. The motion mechanism supports an adjustable speed range of 0–50 mm/s. The imaging unit employs an HIV VISION RGB industrial camera (model MV-CS200–10GC) equipped with a CMOS sensor and a fixed-focus lens with a focal length of 30 mm (model LD-23–0.18X145, manufacturer: Jiangsu Suzhou Youxin Zeda Co.). Together with a ring-shaped constant-illuminance light source (luminous intensity of 183.9 kLux and brightness of 46.341 kcd/m²), the system establishes a standardized imaging environment. Image acquisition operates in a fully automated trigger mode, featuring a closed-loop feedback mechanism for both position coordinates and speed parameters. During image capture, the camera is automatically triggered and transmits image data to a computer via an RJ-45 Gigabit Ethernet interface located at the rear of the incubator. This setup facilitates efficient image editing, dataset construction, and training of instance segmentation models.

The seed growth and cultivation unit (**Figure 1D**) consists of a platform constructed inside the incubator, featuring six fixed positions designed to hold cultivation boxes fabricated via 3D printing. The fixed imaging points for the camera are aligned with these six designated positions. The platform is positioned approximately 1100 mm below the top of the incubator, providing ample vertical space to support the healthy growth of okra seedlings during the seedling stage.

The computer-aided control module (**Figure 1E**) adopts a dual-software collaborative architecture. The camera debugging software is responsible for hardware parameter configuration (such as exposure and resolution), data transmission management, and abnormal event logging. The image processing software manages the entire workflow of data reception, storage, and analysis, establishing a complete data processing pipeline from raw image acquisition to feature extraction.

The human-computer interaction interface (**Figure 1B**) is developed based on the MCGSpro platform, integrating the Mitsubishi FX5U-32MT PLC and the MCGS-Top10s touchscreen. Multi-module data interaction is achieved through MODBUS/TCP and TCP/IP protocols. The main control interface features a dual-mode operation design: the automatic mode supports one-click initiation of the acquisition sequence and parameter presetting, while the manual mode provides fine-grained control options, including motion control (coordinate positioning and speed adjustment), light source management, and shooting interval settings. The real-time status monitoring panel consolidates functions such as equipment operation status display, track coordinate feedback, and limit status indication, ensuring both the safety and convenience of system operation.

### 2.2 Okra seedling dataset construction and image acquisition

**Figure 2:**
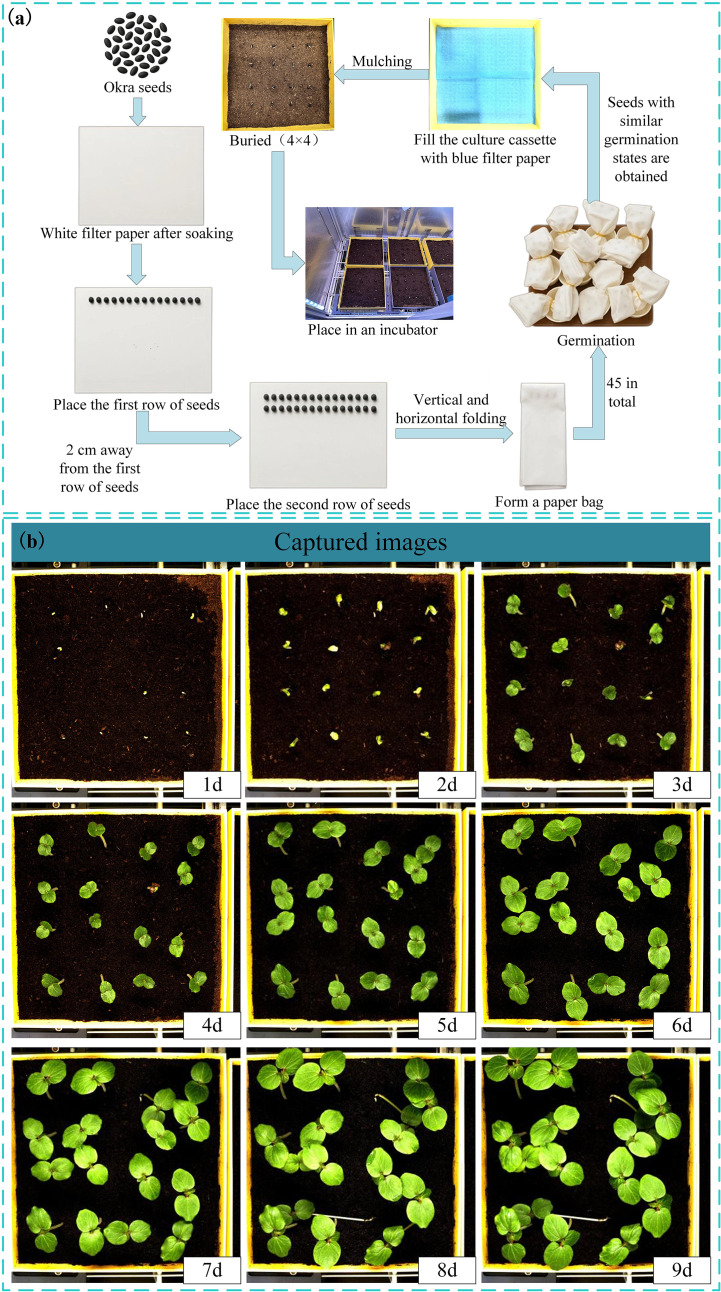
**(a)** Pretreatment of the germination process; **(b)** Collected images of okra seeds at seedling stage.

To train the leaf area and leaf growth rate calculation model for okra seedlings, this study selected 336 red okra seeds (purchased from Shouguang Xinxinran Horticulture Co., Ltd., Shouguang, Shandong, China) that were morphologically intact and uniform in size to construct a seedling monitoring model. Following the germination pretreatment procedure illustrated in **Figure 2a**, a sheet of white filter paper (210 mm × 297 mm) was moistened with deionized water and laid flat on an alcohol-sterilized table. Fifteen okra seeds were evenly spaced 20 mm from the long edge of the paper, followed by another fifteen seeds placed 20 mm apart from the first row. Another sheet of filter paper, moistened halfway, was used to cover the seeds with its wet side, allowing the filter papers between the two rows to adhere to each other. The assembly was rolled by folding the long edge (20 mm) vertically three times and the short edge (40 mm) horizontally three times, secured with a rubber band, and placed into a paper cup containing deionized water. This setup, termed a “cultivation paper pack,” was incubated at 28°C for 24 hours to promote germination. In total, 18 cultivation paper packs were prepared. This process ensured the acquisition of seeds at similar germination states, facilitating subsequent vigor assessment during the seedling growth stage. Subsequently, seeds with similar germination states were neatly and uniformly arranged in a 4×4 grid within six soil-based cultivation trays. These trays were then placed at six fixed positions within the seed growth cultivation module, with an inter-seed spacing of 50 mm. All seeds were then transferred to an incubator maintained at 28 °C under constant temperature and illumination conditions for nine days of full-time seedling monitoring. The experimental timeline commenced when the seeds were placed into the incubator and concluded at the end of the nine-day cultivation period. Utilizing the full-time crop germination vigor monitoring system illustrated in **Figure 1**, images were automatically captured at adjustable 30-minute intervals, yielding a total of 3,456 time-series images. The study employed an HIV VISION RGB industrial camera (model MV-CS200–10GC) equipped with a CMOS sensor and a fixed-focus lens with a 30 mm focal length (model LD-23–0.18X145, manufacturer: Jiangsu Suzhou Youxin Zeda Co.). During each capture cycle, the camera photographed six fixed positions. Additional imaging parameters are detailed in **Table 1**. Representative daily images are shown in **Figure 2b**. According to the criteria established by Martini et al (Martini, 2024), the growth of seeds after soil emergence is classified as the seedling stage. Observations indicated that during the first 24–28 hours of the experiment, leaves had not yet emerged, making it difficult to assess seed vigor (**Figure 2b**). In the later stages, between 24 and 48 hours, substantial leaf overlap was observed (**Figure 2b**), complicating the accurate identification of individual plants. Consequently, 480 images from the initial 24 hours and 960 images from the final 48 hours were excluded, along with 293 images affected by environmental changes, exposure anomalies, or blurring. Ultimately, 1,723 valid images were retained to form the fundamental dataset for model training.

**Table 1:** Test parameters.

Parameter	Numerical value
Solvent	Deionized water
NaCl solution concentration	0(CK),10,20,30,40,50,60 mmol/L
Total number of seeds used	336
The number of seeds in each culture box	16
Germination time	24h
Cultivation time	9d
Temperature	28°C ± 1°C
During the photo-taking break	30min
Number of replicates	3
Total number of photographs taken	3456
Image resolution	1800×1850
Picture format	JPG

**Figure 3:**
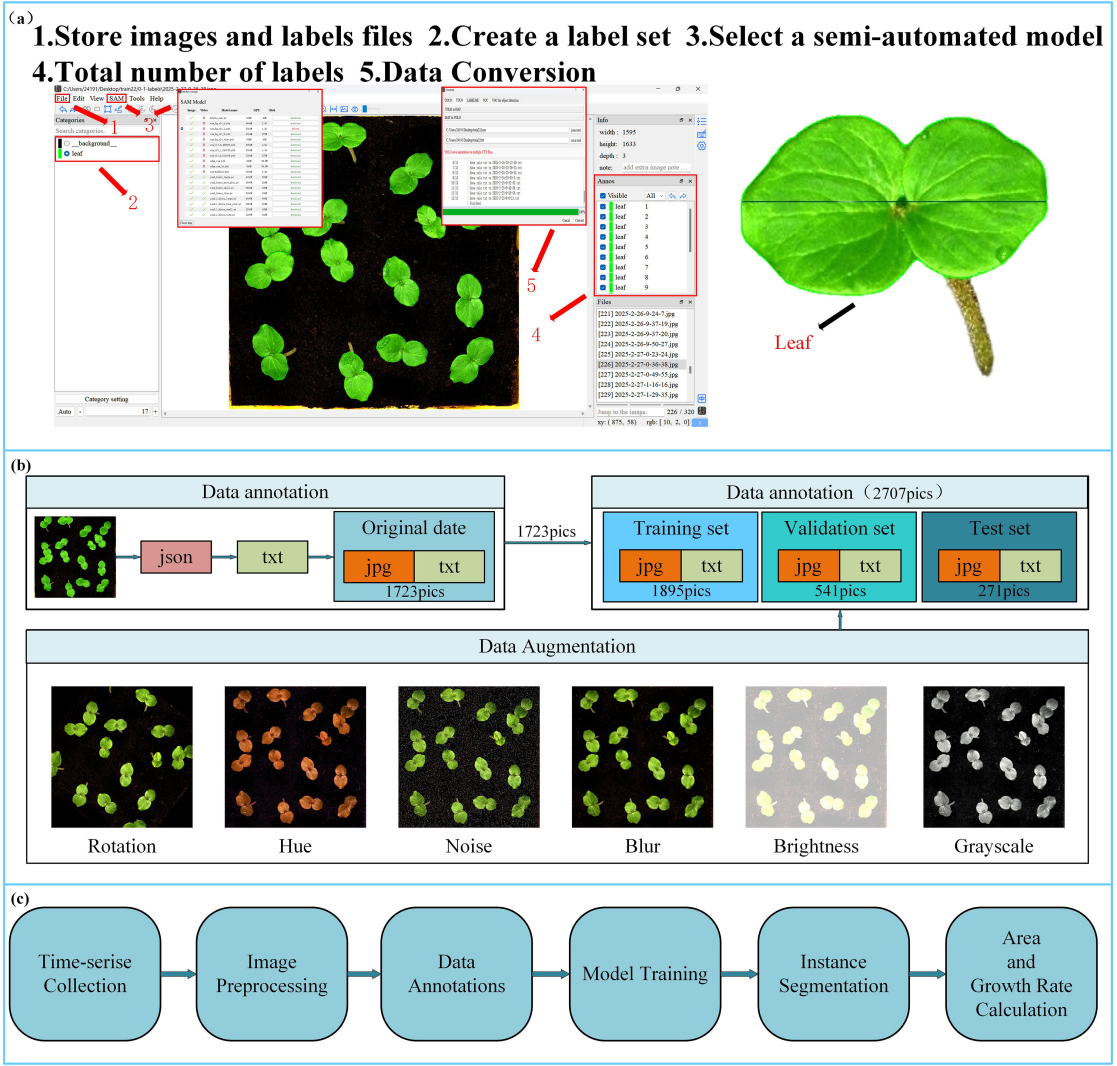
**(a)** ISAT-SAM software annotation process; **(b)** dataset division and dataset enhancement. **(c)** System workflow for time-series data collection, model training, and phenotypic traits calculation.

For dataset annotation, we employed the ISAT-SAM tool (Yateng, 2023) to perform semi-automatic instance segmentation of okra leaves. ISAT-SAM is a semi-automated image annotation tool that integrates Interactive Segmentation Annotation (ISAT) with Meta SAM (Kirillov et al., 2023) (Segment Anything Model). Based on the SAM framework, users can generate high-quality segmentation masks by simply clicking on the target area, significantly reducing the annotation workload. In this study, the annotation was specifically focused on okra leaves, with the label category set as “leaf.” The annotation process is illustrated in **Figure 3a**. The annotation files generated by ISAT-SAM are in JSON format, which does not directly meet the requirements for instance segmentation model training. Therefore, we utilized the data conversion tool provided by ISAT-SAM to extract key parameters such as image resolution, category identifiers, and bounding box coordinates, and normalized the pixel coordinates to generate a TXT file compatible with the YOLO training standard.

According to the occlusion levels defined by Paul (Paul et al., 2024b; Paul and Machavaram, 2025), this study considers two levels of occlusion: No Occlusion and Leaves Occlusion. The imaging was conducted under constant illumination conditions, with a light intensity of 183.9 kLux and a luminance of 46.341 kcd/m². This setup ensured uniform lighting across the entire dataset, facilitating consistent feature extraction for subsequent model training and evaluation. To enhance the model’s anti-overfitting capability and generalization across different scenes, multi-dimensional data augmentation strategies were employed (**Figure 3b**), including rotation (within the range of -45° to +45°), hue adjustment (between -180° and +180°), brightness adjustment (within -90% to +90%), blur (up to 5.7px), and noise (up to 8.26% of pixels). This augmentation process resulted in a total of 2707 datasets. After splitting the dataset at a ratio of 70:20:10, a complete dataset comprising 1,895 training images, 541 validation images, and 271 test images was obtained (the partitioning process is illustrated in **Figure 3b**). This proportion ensures an appropriate and balanced distribution of samples across subsets, which is critical for building robust models and performing reliable evaluations. Such a division strategy is commonly employed in instance segmentation tasks (Hong et al., 2025) and seed germination object detection studies (Tian et al., 2025). The partition process is illustrated in **Figure 3b**. By simulating variations in imaging conditions and viewing angles, this approach significantly enhances the model’s adaptability to complex experimental environments. Throughout all image processing and annotation conversion stages, the original resolution and spatial accuracy of the annotations were preserved to ensure the reliability of the object detection model training.

To preliminarily validate the usability of the constructed dataset, we trained the YOLOv11 model using the complete dataset. The trained model was then employed to perform inference on randomly selected okra leaf images. As illustrated in **Figure 1F**, the model successfully generated object detection bounding boxes and corresponding segmentation mask outputs. These results demonstrate that the dataset possesses good usability and is suitable for supporting subsequent model training and performance evaluation.

**Figure 3C** illustrates the overall workflow of this study, including time-series data collection, image preprocessing, annotation, model training, instance segmentation, and leaf area, growth rate calculation.

### 2.3 Okra seedling growth experiment under salt stress

To investigate the potential impact of salt stress on the vigor of okra seedlings, we designed a full-time monitoring experiment under soil culture conditions. In this experiment, sodium chloride (NaCl) solution was primarily used to simulate a salt stress environment (Rewald et al., 2015), with six different NaCl solution concentrations (ranging from 10 to 60 mmol/L) in 10 mmol/L increments. The concentration range was determined based on two key considerations. First, preliminary experiments indicated that the sensitivity threshold of okra seedlings to NaCl is approximately 10 mmol/L, with higher concentrations (≥20 mmol/L) significantly inhibiting leaf expansion. Second, Ullah et al. (2024) reported that even under low-salinity conditions (50 mM NaCl), the seedling vigor of most okra cultivars was markedly reduced (Ullah and Jan, 2024). Deionized water was used as the control group (CK) to compare differences in leaf area and leaf growth rate between the treatment groups and normal water conditions. To minimize experimental variability, two replicate experiments were conducted simultaneously, with a total of 21 culture cassettes. During the experiment, 250 ml of the corresponding solution was sprayed into each culture box every 12 hours. **Table 1** presents the remaining experimental parameters. In this study, based on the full-time seedling stage leaf image data collected under salt stress conditions, a deep learning instance segmentation model was developed to systematically analyze the spatiotemporal dynamics of okra seedling growth vitality indices (leaf area and leaf growth rate) under different salt stress levels. The aim was to establish an intelligent evaluation system for okra seedling vitality using computer vision techniques.

### 2.4 Calculation model of leaf area growth rate

#### 2.4.1 Model training conditions and hyperparameter settings

The processor used in this experiment was a 12th Gen Intel^®^ Core™ i5-12500H (2.50 GHz), with Windows 11, operating system and an NVIDIA GeForce RTX 3050 Ti graphics card. The deep learning framework utilized was PyTorch 2.6.0 (developed by Facebook Artificial Intelligence Research, FAIR), running in a virtual environment created via Anaconda3. Python 3.11 (developed by the Python Software Foundation, PSF) and CUDA version 12.6 (developed by NVIDIA) were employed for training the deep learning model on okra seedling process images. The remaining environment configurations are listed in **Table 2**.

**Table 2:** Model training environment.

Environmental parameters	Parameter description
Virtual Environment Manager	Anaconda3
Virtual Environment Name	Okra-leaf
Development language	Python3.11
Deep Learning Framework	Pytorch2.6.0
Image processing library	Torchvision 0.21.0
GPU Acceleration Library	Cuda 12.6
Numerical Calculation Library	NumPy 2.2.4
Cartography	Matplotlib 3.10.1

During training, hyperparameters were carefully fine-tuned to optimize model performance, with the key parameters summarized in **Table 3**. These include the learning rate for convergence speed and stability, the number of warm-up epochs to prevent gradient oscillation due to an initially high learning rate, and weight decay to mitigate overfitting and regulate model complexity. These settings ensured that the YOLOv11-HSECal model was effectively trained for accurate okra instance segmentation. To ensure fairness and comparability across experiments, all models were trained from scratch without the use of pretrained weights.

**Table 3:** Model training hyperparameter settings.

Hyperparameter name	Specific information
Epoch	100
Batch size	4
Optimizer	SGD
Image Size	640×640
Initial Learning Rate	1×10^-2^
Final Learning Rate	1×10^-4^
Weight-Decay	5×10^-4^
Warmup-epochs	3

The model was trained using the Stochastic Gradient Descent (SGD) optimizer (Ketkar, 2017), which is known for its stability and generalization capability, especially suitable for agricultural imaging scenarios involving high-resolution inputs and a limited number of object instances. The initial learning rate was set to 1×10^-2^, and a warm-up strategy was adopted, linearly increasing the learning rate during the first 3 epochs to avoid early-stage training instability. A StepLR scheduler (Wen et al., 2023) was then applied to reduce the learning rate by a factor of 0.5 every 20 epochs, gradually decaying it to 1×10^-4^ to ensure smooth convergence in the later stages.

To further suppress overfitting, a weight decay of 5×10^-4^ was introduced as an L2 regularization term in the optimizer, which constrains the magnitude of model weights and improves generalization. This parameter was selected based on the official YOLO recommendations and refined through pre-experiments within the 1×10^-4^ to 1×10^-3^ range. Results indicated that setting the weight decay to 5×10^-4^ achieves a good trade-off between convergence speed and detection accuracy.

#### 2.4.2 Construction of YOLOv11-HSECal model

**Figure 4:**
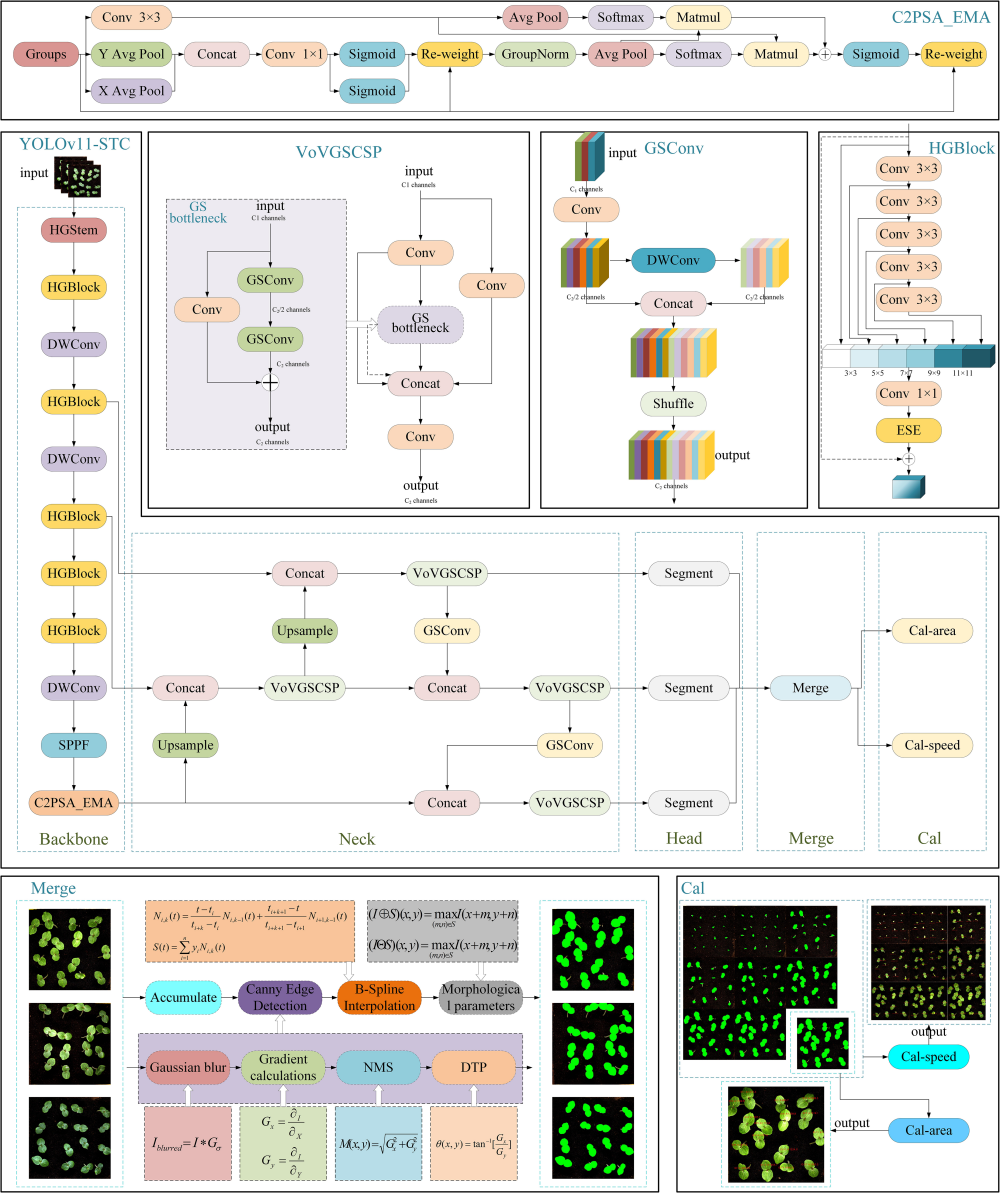
Improved YOLOv11-HSECal model network structure.

In the early seedling stage, when okra first emerges from the soil, the target Leafs are relatively small and often exhibit irregular edge contours—referring to boundaries composed of multiple curved and serrated segments rather than smooth lines or arcs—which substantially increases the complexity of segmentation tasks. These characteristics pose significant challenges for the YOLOv11-seg model in accurately detecting and segmenting such fine-grained features. Although YOLOv11-seg has demonstrated strong performance in object detection and instance segmentation across various applications, it still presents certain limitations in segmentation accuracy (mAP), computational load (FLOPs), and model parameter size. To address these shortcomings, this study proposes a series of optimizations to the original model. Specifically, the YOLOv11-seg backbone was replaced with HGNetv2 from the RT-DETR framework (Zhao et al., 2024); the Neck component was substituted with the lightweight Slim-Neck (Li HL. et al., 2024); and the EMAttention mechanism (Ouyang et al., 2023) was introduced to enhance feature representation. Furthermore, two additional modules—Merge and Cal—were integrated to construct the proposed YOLOv11-HSECal model, as illustrated in **Figure 4**. These modifications significantly improve the model’s segmentation capability for small and irregular targets, increase segmentation precision, and reduce both FLOPs and parameter count. The enhanced model also facilitates the accurate computation of leaf area and growth rate, thereby improving the model’s real-time performance and deployment potential on resource-constrained edge devices. The specific improvements are as follows:

1. The backbone of the YOLOv11-seg model was replaced with HGNetv2, the backbone architecture of the RT-DETR model, to address the original model’s limited ability to comprehend and process complex scenes. HGNetv2 is a graph neural network (GNN)-based architecture specifically designed to handle complex data with hierarchical structures. By constructing a multi-level graph topology and integrating it with graph convolution operations, HGNetv2 effectively captures both node relationships and global contextual information across multiple scales. This design substantially enhances the model’s capacity to tackle intricate visual tasks. The hierarchical feature propagation mechanism enables the fusion of local and global representations, thereby improving the performance and robustness of tasks such as node classification, graph classification, and graph generation. Integrating HGNetv2 into the YOLOv11-seg framework enhances segmentation accuracy while simultaneously reducing model parameters and computational complexity (FLOPs) to a certain extent.
2. The Slim-Neck feature fusion module was introduced to replace the original neck component, aiming to optimize the feature fusion process and enhance segmentation accuracy for small targets and irregular leaf contours. Slim-Neck improves the efficiency of feature integration by incorporating the VoV-GSCSP module and GSConv (a hybrid convolution module), thereby reducing redundant computations. In this architecture, feature maps at various scales are first processed through the GSConv module. These processed maps are then fused with feature maps from other scales using bilinear interpolation for upsampling, followed by concatenation operations. The resulting fused maps are further refined through another pass of GSConv, followed by additional feature extraction and integration using the VoV-GSCSP module. This design enables more effective multi-scale feature representation, particularly enhancing the detection performance for small-scale objects and improving the model’s robustness and accuracy under complex visual conditions.
3. The EMAttention mechanism was introduced to replace the original C2PSA attention module in YOLOv11-seg, aiming to enhance the model’s object detection and segmentation performance in complex backgrounds and scenarios with overlapping objects. EMA (Efficient Multi-scale Attention) is a novel attention mechanism designed to improve feature representation while reducing computational overhead. It captures both short-range and long-range dependencies within feature maps through a multi-scale attention architecture. Unlike conventional attention mechanisms, EMA avoids dimensionality reduction, thereby preserving rich channel information and strengthening spatial feature aggregation. Additionally, EMA employs parallel sub-networks with 1×1 and 3×3 convolutional kernels to aggregate multi-scale information from feature groups. It also captures pixel-level pairwise relationships through cross-spatial learning, resulting in more refined spatial feature distributions and improved contextual understanding of images. Experimental results demonstrate that integrating EMA significantly enhances the detection and segmentation accuracy of the YOLOv11-seg model.
4. The Merge module was incorporated to address the issue of multiple masks resulting from the segmentation of numerous okra leaves within a single image. Following instance segmentation, each image yields multiple leaf masks, with the segmentation output containing several mask attributes. To integrate these, the Accumulate module iteratively processes each individual mask by converting it to the uint8 format, resizing it to match the original image dimensions, and overlaying the masks onto the original image to generate a unified merged mask containing all segmentation information. A thresholding operation is then applied to the merged mask to ensure pixel values fall within the range of (0, 255). The final merged mask is saved as a high-resolution image file named mask.jpg (1800×1850 pixels). Concurrently, binary images and bounding boxes corresponding to each segmented leaf region are extracted, and the central coordinates (Cx, Cy) and pixel counts of individual masks are output. To further enhance the quality of the instance segmentation masks for okra leaves, a series of image processing techniques were applied, including Canny edge detection, B-spline interpolation smoothing, and morphological operations such as dilation and erosion (Unser et al., 1993; Ding and Goshtasby, 2001; Kang et al., 2016). The integration of these methods significantly improves the precision and smoothness of the segmentation masks, thereby enhancing the accuracy of leaf area calculation and the reliability of leaf growth tracking over time.
5. The Cal module was introduced to compute leaf area and leaf growth rate based on the pixel data and mask images transmitted from the Merge module. This module is subdivided into Cal-area and Cal-speed, depending on the nature of the dataset. For individual images or datasets without temporal continuity, the Cal-area module calculates the leaf area for each sample. In contrast, for datasets representing full time-series growth sequences, the Cal-speed module is employed to compute the real-time growth rate of each leaf, while simultaneously outputting corresponding leaf area values. To enable accurate tracking of individual leaves across different time points, a cross-frame label tracing method was developed. Each leaf is assigned a unique identifier (ID), which is maintained throughout the sequence. In the initial frame, 16 leaves fixed within the image are arranged in a 4×4 grid. Based on the vertical coordinate (Cy) of the leaf centroid, the image is divided into four horizontal rows. Within each row, leaves are sorted by their horizontal centroid coordinate (Cx), and assigned IDs sequentially from left to right, ranging from 0 to 15. For subsequent frames, newly detected leaves are matched to previously identified ones by comparing their segmentation masks to those from the preceding frame. The Intersection over Union (IoU) metric (Cheng et al., 2021) is employed as the matching criterion. If the maximum IoU for a candidate leaf exceeds a predefined threshold (e.g., 0.5), it is deemed to represent the same leaf, and the corresponding ID is inherited. This method ensures continuous and reliable tracking of individual leaves across all frames, facilitating precise analysis of temporal growth patterns.

In the actual model training process, the Merge and Cal modules are not directly involved during the training phase; therefore, they have a negligible impact on key model metrics such as segmentation accuracy (mAP), the number of model parameters, and FLOPs.

#### 2.4.3 Okra seedling vigor evaluation index

The leaf area and leaf growth rate during the seedling stage of okra are key indicators directly reflecting the vigor of okra seeds and the growth status of seedlings (Al-Musawi and Al-Moussawi, 2020). The actual leaf area is calculated using the Cal-area module based on Equations 1, 2, with image resolution set at 1800 × 1850 pixels and an actual physical length of 250 mm for the image. The corresponding formulas are as follows:


(1) $$\begin{array}{c} \;Pixel_{Area\;}\left(mm^{2}\right)={\left(\frac{250}{\;Img_{Width\;}}\right)}^{2}\; \end{array}$$

(2) $$\begin{array}{c} Leaf_{Area}\;\left(mm^{2}\right)=\;Pixel_{Area\;}\times \;Sum_{Pixel\;}\; \end{array}$$

where $Pixel_{Area\;}$ denotes the actual area represented by each pixel; $Img_{Width\;}$ refers to the total number of pixels along the image boundary; $Leaf_{Area}$ represents the actual area of each individual leaf; and $Sum_{Pixel\;}$ corresponds to the total number of pixels in the two-dimensional Boolean matrix obtained through segmentation (Benhacine et al., 2019).

The full time-series dataset utilized in this study incorporates timestamps in the format “YYYY-MM-DD-HH-MM-SS”. The Cal-speed module extracts temporal information from these timestamps using regular expressions and computes the time interval (unit: hours) between successive frames. Once the leaf area for each dataset is obtained, the Cal-speed module calculates the real-time growth rate of okra leaves based on Equation 3, enabling the analysis of growth rate variations under different salt stress conditions. The formula is presented as follows:


(3) $$\begin{array}{c} Growth\;Rate=\frac{Area_{current}-Area_{previous}}{t_{i}-t_{i-1}}\; \end{array}$$

where *Growth Rate* denotes the leaf growth rate, while *Area_current_* and *Area_previous_* represent the leaf areas in the current and previous frames, respectively. *t_i_* and *t_i-1_* represent the timestamps of the current and previous frames, respectively. Notably, the time interval is not limited to consecutive frames; it can also represent any user-defined time window, allowing for flexible analysis of okra growth rates across different temporal scales. Accordingly, we propose leaf area and leaf growth rate as quantitative indicators for evaluating the growth vigor of okra seedlings.

#### 2.4.4 Evaluation indicators of okra leaf segmentation model

Instance segmentation models are typically evaluated using six key metrics: precision (P), recall (R), and average precision (mAP50and mAP50–95) to assess model accuracy, along with the number of parameters (Params) and floating-point operations per second (FLOPs) to evaluate model complexity (Khanam and Hussain, 2024).

Average precision is employed to evaluate the accuracy of the model in recognizing and segmenting okra leaves. A higher threshold in mAP indicates a greater overlap between the predicted elements (bounding box and mask) and the ground truth target, thereby providing a more stringent assessment of the model’s capability to precisely localize objects. Conversely, a lower threshold emphasizes the model’s ability to determine the presence of a target, regardless of localization precision. This relationship can be quantitatively described by Equations 4–8.


(4) $$\begin{array}{c} P=\frac{TP}{TP+FP}\; \end{array}$$

(5) $$\begin{array}{c} R=\frac{TP}{TP+FN}\; \end{array}$$

(6) $$\begin{array}{c} AP=\int_{0}^{\;1}P\left(R\right)dR\; \end{array}$$

(7) $$\begin{array}{c} mAP_{50}=\frac{1}{n_{c}}\int_{0}^{\;1}P\left(R\right)dR\; \end{array}$$

(8) $$\begin{array}{c} mAP_{50-95}=avg\left(mAP_{i}\right),i=50,55,\ldots ,95\; \end{array}$$

where *TP* denotes true positives, where the leaf segmentation model correctly detects and segments actual instances of okra seedling leaves. *FP* represents false positives, referring to instances where the model incorrectly detects or segments non-existent okra seedling leaves (e.g., mistaking soil texture, culture box edges, or light-induced noise as leaf regions) or produces segmentation results that do not meet the required criteria. *FN* indicates false negatives, where the model fails to detect or segment real okra seedling leaves. In Equation 6, AP denotes the average precision obtained by integrating the area under the Precision-Recall curve. Here, P(R) represents the precision at a given recall level R, and dR is the integration variable corresponding to an infinitesimal change in recall. A higher average precision reflects better overall performance of the model in accurately detecting and segmenting okra leaves.

The lightweight nature and computational complexity of the model are assessed using the number of parameters and floating-point operations per second (FLOPs), as defined by Equations 9, 10, respectively. In these equations, K^2^ denotes the area of the convolutional kernel, *H* × *W* represents the height and width of the input feature map, *C*_in_ is the number of input channels, and *C*_out_ is the number of output channels.


(9) $$\begin{array}{c} FLOPs=2\times H\times W\left(C_{in}K^{2}+1\right)C_{out}\; \end{array}$$

(10) $$\begin{array}{c} Params=C_{in}\times K^{2}\times C_{out}\; \end{array}$$

Parameters focus on measuring the complexity and storage cost of the model, affecting the training difficulty and the risk of overfitting. FLOPs measure the computational cost and operational efficiency of the model, and determine the hardware suitability and real-time performance. Therefore, on the premise of ensuring accuracy, the smaller the two, the more cost-effective the model (Wan et al., 2023).

## 3 Results

### 3.1 Analysis of training loss for YOLOv11-HSECal

**Figure 5:**
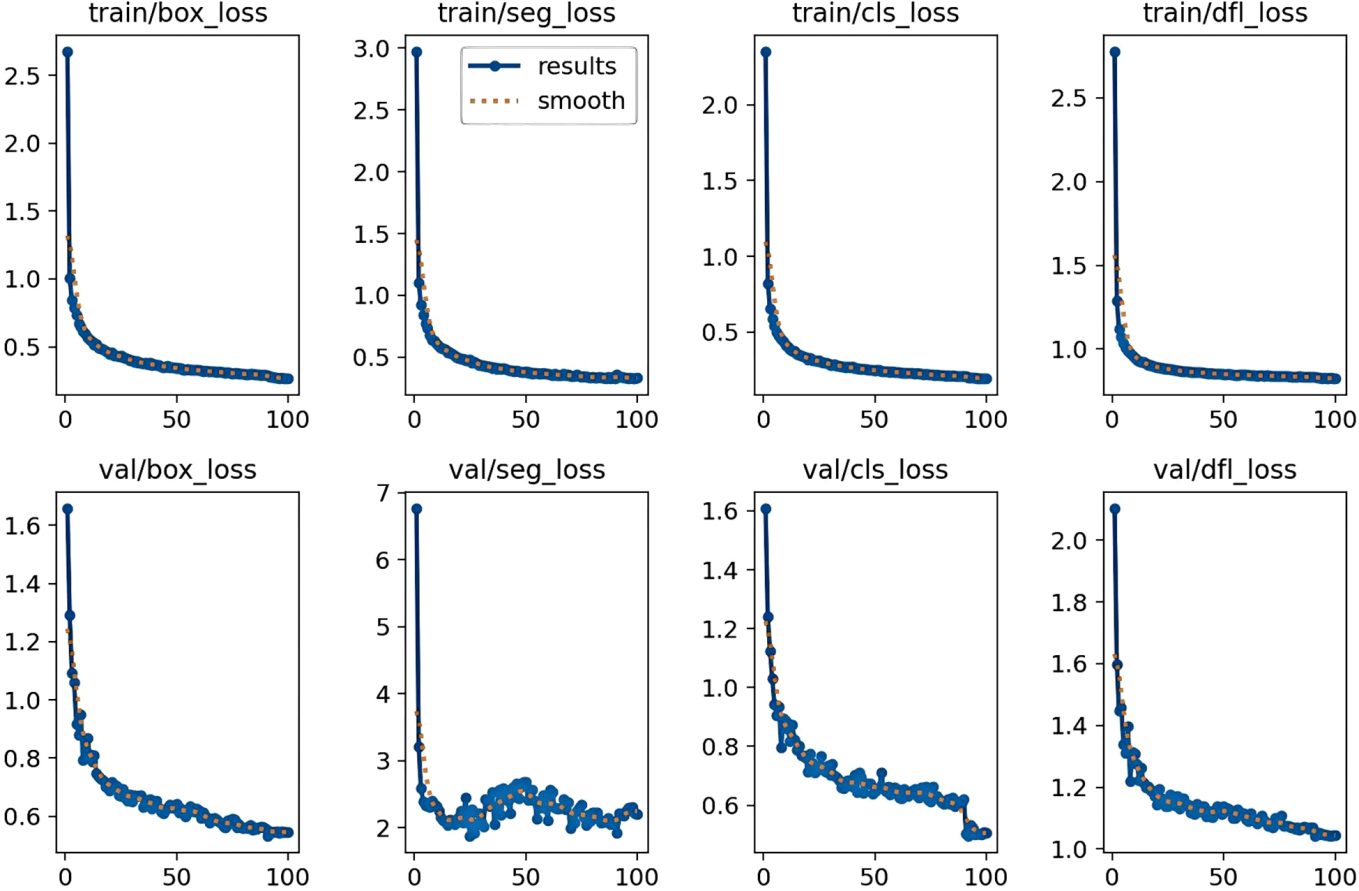
Training loss curves during custom training of YOLOv11-HSECal model.

In neural networks, the term “loss” refers to a measure of prediction inaccuracy. The primary metrics used to indicate training error in instance segmentation include box loss, segmentation loss, classification loss, and Distribution Focal Loss (DFL), as illustrated in **Figure 5**. Box loss quantifies the algorithm’s ability to accurately localize object centers and the precision of predicted bounding boxes containing the objects. Segmentation loss measures the discrepancy between predicted pixel-level labels and ground truth masks. Classification loss evaluates the accuracy of the predicted object classes. Distribution Focal Loss (DFL) is specifically designed to mitigate class imbalance during network training, which occurs when certain classes are overrepresented (Paul and Machavaram, 2025).

The training box loss exhibited a sharp decline around the 2nd epoch, followed by a steady decrease until approximately the 55th epoch, and stabilized near the 85th epoch with a minimum value of 0.266. The other three losses followed similar trajectories, reaching minimum values of 0.328, 0.194, and 0.824, respectively. The initial rapid decline in all losses is attributed to hyperparameter tuning, while the stabilization around the 85th epoch validates the decision to terminate training at the 100th epoch.

The validation loss curves demonstrated trends similar to the training loss but with greater fluctuations, indicating the model’s adaptability to the validation dataset. Validation box loss showed a sharp decrease during early epochs, followed by irregular fluctuations and eventually stabilized around the 78th epoch with a minimum of approximately 0.533. Validation segmentation loss dropped rapidly within the first four epochs, fluctuated intensely between the 8th and 63rd epochs, and stabilized near the 92nd epoch with a minimum value of about 1.919. Validation classification loss sharply declined in the first 10 epochs, oscillated around the 20th epoch, then gradually decreased before stabilizing after a sharp drop at the 91st epoch, with a minimum near 0.496. Validation DFL loss exhibited a similar pattern, with overall stabilization accompanied by fluctuations within a range and a minimum value of approximately 1.042. The fluctuations in validation loss reflect the model’s sensitivity to unseen data, yet the overall downward trend indicates improved generalization performance, consistent with the stable trend observed in training loss.

### 3.2 Longitudinal comparison of YOLOv11-HSECal with iterative versions of YOLO series instance segmentation models

**Figure 6:**
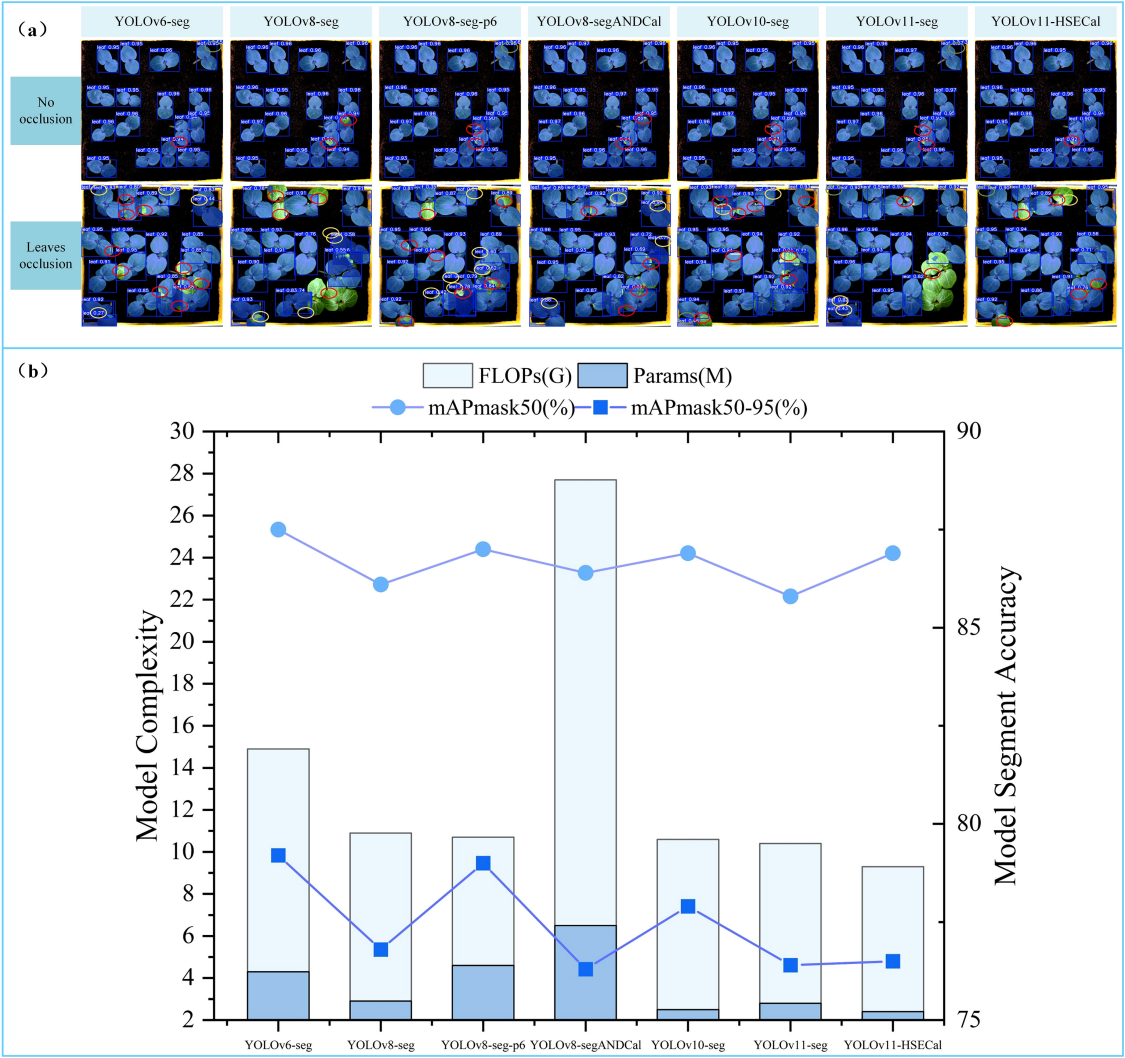
**(a)** Comparative analysis of segmentation performance across models **(b)** Longitudinal comparative analysis of the performance indicators of the YOLO model.

To comprehensively validate the performance advantages of the proposed model in okra leaf detection and segmentation tasks, we selected several other YOLO-series instance segmentation models as benchmarks, including YOLOv6-seg, YOLOv8-seg, YOLOv8-seg-p6, YOLOv8-segANDCal, YOLOv10-seg, and YOLOv11-seg. Systematic experiments were conducted under unified configurations and hyperparameter settings. **Figure 6a** presents the segmentation results of these seven models under two occlusion levels during the okra seedling stage: No Occlusion and Leaves Occlusion. “No Occlusion” indicates that okra leaves are fully visible without overlap, whereas “Leaves Occlusion” refers to scenarios where leaves overlap and cause mutual occlusion. In the figure, yellow indicates duplicate segmentation, green represents erroneous segmentation, and red denotes incomplete segmentation. Although YOLOv8-seg and YOLOv8-seg-p6 both belong to the YOLOv8 family, they differ significantly in network architecture. YOLOv8-seg is constructed based on three backbone feature outputs corresponding to 8×, 16×, and 32× downsampled feature maps (denoted as P3/8, P4/16, and P5/32), offering a favorable balance of detection efficiency and model compactness. Conversely, YOLOv8-seg-p6 further incorporates a P6 layer (P6/64), a 64× downsampled deep feature map that enhances semantic representation and improves recognition of small targets, albeit with increased computational complexity and parameter count. Under the No Occlusion condition, models rarely produced erroneous segmentations, with YOLOv11-HSECal exhibiting only one instance of incomplete segmentation. Under Leaves Occlusion, segmentation accuracy declined markedly across models; however, YOLOv11-HSECal demonstrated superior robustness, with only one duplicate and one erroneous segmentation instance, and relatively fewer incomplete segmentations compared to other models. These results indicate that YOLOv11-HSECal achieves outstanding segmentation performance.

Wu et al. (2024) utilized an improved YOLOv8-segANDCal model to estimate soybean root length, enhancing local feature extraction of soybean radicles via the SegNext_Attention mechanism. Nonetheless, its architecture primarily targets linear structures (e.g., roots), limiting adaptability to irregular leaf morphologies. We thus conducted a comparative analysis between YOLOv11-HSECal and YOLOv8-segANDCal. Performance evaluations on datasets featuring No Occlusion and Leaves Occlusion conditions (see **Figure 6a**) revealed that the custom-trained YOLOv11-HSECal outperforms YOLOv8-segANDCal in precision, recall, and F1 score (**Table 4**). Overall, YOLOv11-HSECal demonstrates superior segmentation capability compared to YOLOv8-segANDCal.

**Table 4:** Comparison of the performance of the two models at different occlusion of okra.

Models	Occlusion Level	Precision	Recall	F1-score	Average inference time/peduncle (ms)
YOLOv11-HSECal	No occlusion	0.99	0.98	0.98	7.5
	Leaves occlusion	0.88	0.85	0.87	9.1
YOLOv8-segANDCal	No occlusion	0.98	0.96	0.97	11.4
	Leaves occlusion	0.83	0.79	0.82	13.9

According to the experimental results shown in Figure X(B), the proposed model achieves superior lightweight performance while maintaining high segmentation accuracy. Specifically, the segmentation mask mAP50 and mAP50-95 reached 86.9% and 76.5%, respectively, while the FLOPs and parameter count were reduced to 9.3G and 2.4M. Here, G denotes the number of giga floating-point operations (GFLOPs), and M represents the number of million trainable parameters contained in the model. Compared to YOLOv11-seg, our model improved mAP50 by 1.1%, reduced FLOPs by 0.6%, and decreased the number of parameters by 14.1%. Although YOLOv6-seg and YOLOv8-seg-p6 exhibited slightly higher segmentation accuracy, their computational complexity increased significantly, with FLOPs and parameter counts nearly doubling those of our model.

These comparative experiments demonstrate that our model achieves the optimal balance between segmentation accuracy and model lightweight design. The average precision of the segmentation mask directly influences the accuracy of leaf area and growth rate calculations. Meanwhile, a high number of model parameters and FLOPs imposes substantial demands on hardware resources. In contrast, our model significantly reduces hardware requirements while maintaining a high segmentation accuracy after lightweight optimization. This makes it particularly well-suited for practical agricultural applications, where computational efficiency and accuracy are both critical at lab or field level.

### 3.3 Horizontal comparison between YOLOv11-HSECal and state-of-the-art instance segmentation models such as grounded SAM

**Figure 7:**
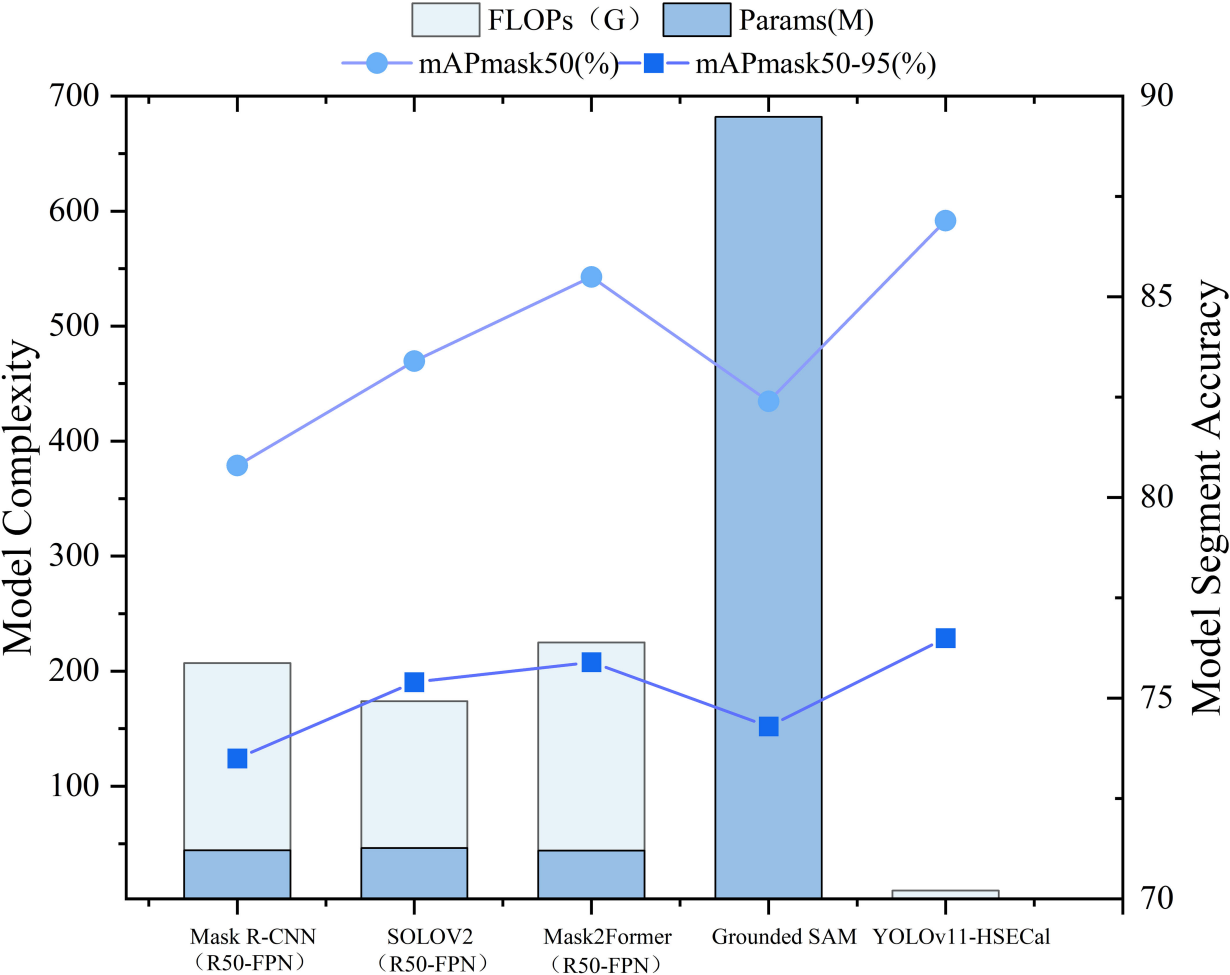
Side-by-side comparative analysis of other advanced model performance indicators.

Ayan Paul et al. (Paul et al., 2024a) previously conducted comparative experiments between YOLOv9c-seg and the Grounded SAM model for instance segmentation of pepper pedicels. Building upon this, we aim to further evaluate the strengths and weaknesses of YOLO-based models in comparison to Grounded SAM for instance segmentation tasks. To validate the horizontal effectiveness of our proposed YOLOv11-HSECal model, we also conducted comparative analyses with other state-of-the-art but computationally intensive segmentation frameworks, including Mask2Former (R50-FPN) (Cheng et al., 2022), SOLOV2 (R50-FPN) (Wang et al., 2020), and Mask R-CNN (R50-FPN) (He et al., 2017). All models were trained and tested under identical experimental settings to ensure a fair comparison, as shown in **Figure 7**.

Our results indicate that, although models such as Grounded SAM, Mask R-CNN (R50-FPN), SOLOV2 (R50-FPN), and Mask2Former (R50-FPN) have demonstrated solid performance in many previous segmentation tasks, they offer no significant accuracy advantage in our case. Moreover, they exhibit substantially higher model sizes and computational costs. These findings highlight the superiority of YOLOv11-HSECal in achieving competitive segmentation performance with greater computational efficiency.

### 3.4 Ablation experiment of YOLOv11-HSECal, a seedling leaf segmentation model of okra

**Figure 8:**
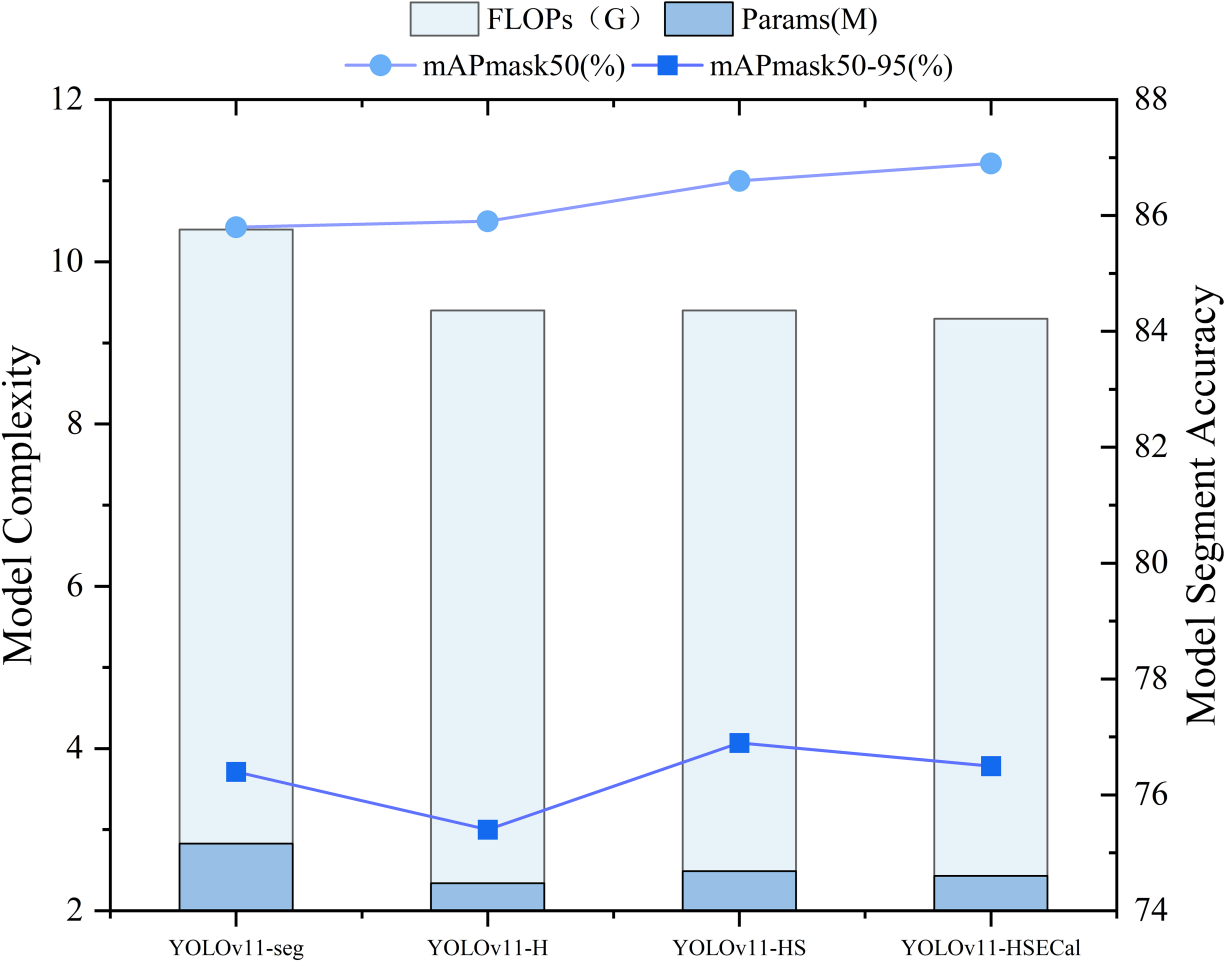
Comparison of YOLOv11-HSECal model ablation experiment performance indicators on the validation sets.

To evaluate the effectiveness of the aforementioned improvements, a series of ablation experiments were conducted. Four model variants were assessed on the same validation dataset. As illustrated in **Figure 8**, replacing the backbone of YOLOv11-seg with HGNetv2 resulted in the YOLOv11-H model, which maintained comparable accuracy while achieving a 17% reduction in model parameters and a 9.6% decrease in FLOPs, thereby demonstrating the effectiveness of the lightweight design. Subsequent replacement of the Neck component with the Slim-Neck module in YOLOv11-H led to the YOLOv11-HS model. Although this increased the number of parameters by 6.03%, it yielded a 0.7% improvement in mAP50, with no additional increase in FLOPs. Building upon YOLOv11-HS, the introduction of the EMAttention mechanism further enhanced detection performance, increasing mAP50 by an additional 0.3%, while reducing parameters and FLOPs by 2.25% and 1.06%, respectively. These findings indicate that, compared to YOLOv11-seg, the proposed YOLOv11-HSECal model significantly reduces model complexity while achieving a 1.1% gain in mAP50. As shown in **Figure 8**, the YOLOv11-HSECal model demonstrates enhanced accuracy in okra leaf segmentation while maintaining computational efficiency, making it more suitable for deployment on resource-constrained hardware platforms.

### 3.5 Accuracy evaluation of the model algorithm

**Figure 9:**
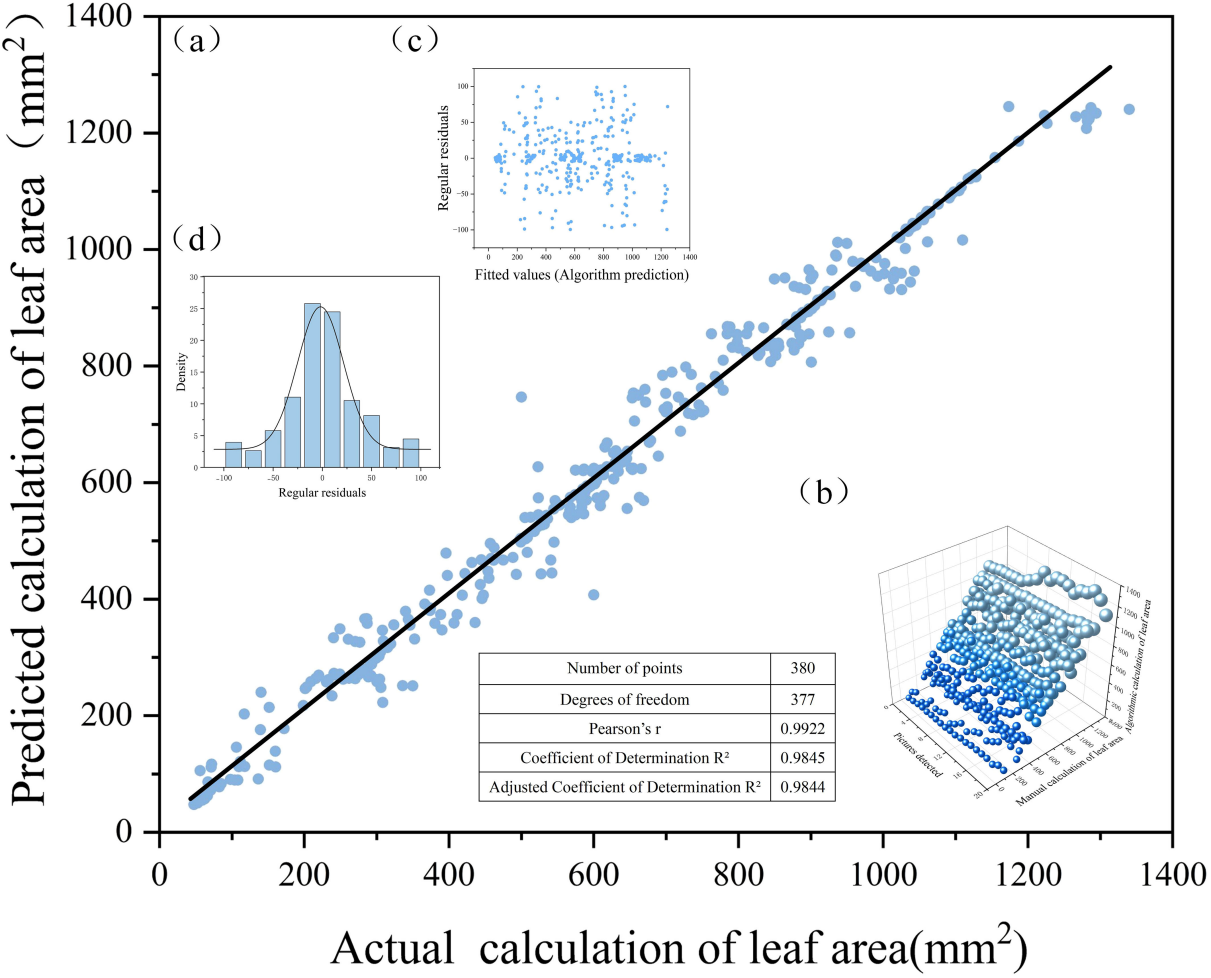
Correlation analysis between algorithmic detection values and manual measurements: **(a)** fitted straight line; **(b)** manual and algorithmic count statistics; **(c)**scatter plot of residuals; and **(d)** normal distribution plot.

In this study, the leaf area of okra at the seedling stage was used as an accuracy benchmark. To validate the reliability of this metric, a total of 380 images were randomly selected from those captured in the culture box, and a plant exhibiting relatively complete and consistent growth morphology was tracked. The leaf area of this target plant was calculated using both manual and algorithm-based methods. The algorithmic procedure involved three steps: first, instance segmentation was performed using the YOLOv11-HSECal model to generate the mask image; second, the Merge module was employed to refine and optimize the mask, yielding accurate pixel-level data; and finally, the Cal module was used to track the target plant across images by matching its unique ID number, thereby computing the algorithm-derived leaf area. Corresponding manual measurements were conducted based on the same ID. To assess the correlation, agreement, and error characteristics between the manual and algorithmic measurements, regression fitting plots, residual plots, and normal distribution plots were generated, as illustrated in **Figure 9**.


(11) $$\begin{array}{c} r=\frac{Cov\left(X,Y\right)}{\sqrt{Var\left|X\right|\cdot Var\left|Y\right|}}\; \end{array}$$

(12) $$\begin{array}{c} R^{2}=1-\frac{\sum_{i=1}^{n}{\left(y_{i}-{\hat{y}}_{i}\right)}^{2}}{\sum_{i=1}^{n}{\left(y_{i}-\bar{y}\right)}^{2}}\; \end{array}$$

(13) $$\begin{array}{c} Adjusted\;R^{2}=1-\left(\frac{1-R^{2}}{n-k-1}\right)\left(n-1\right)\; \end{array}$$

(14) $$\begin{array}{c} y=ax+b\; \end{array}$$

(15) $$\begin{array}{c} a=\frac{\sum_{i=1}^{n}(x_{i}-\bar{x})\left(y_{i}-\bar{y}\right)}{\sum_{i=1}^{n}{\left(x_{i}-\bar{x}\right)}^{2}}\; \end{array}$$

(16) $$\begin{array}{c} y=1.0078x-6.6564\; \end{array}$$

Here, Cov (*X*, *Y*) denotes the covariance between variables *X* and *Y*, while Var|*X*| and Var|*Y*| represent the variances of *X* and *Y*, respectively. The parameter a corresponds to the slope of the fitted regression line, and b represents the intercept on the Y-axis.

**Figure 9A** presents the distribution of leaf area measurements obtained through manual annotation and algorithmic calculation. To further validate the consistency between model predictions and manual measurements, a regression fitting plot was generated by setting manually calculated leaf area as the horizontal axis and the model-predicted leaf area as the vertical axis. The correlation analysis yielded a Pearson correlation coefficient of r = 0.9922 (as defined in Equation 11), indicating a very strong linear relationship between the two variables. The corresponding coefficient of determination was calculated as R² = 0.9845 (Equation 12), suggesting that approximately 98.45% of the variance in leaf area can be explained by the model’s predictions, demonstrating excellent fitting performance. Considering the effects of sample size and the number of independent variables, the adjusted coefficient of determination was further calculated as R² = 0.9844 (Equation 13), which remains at a high level. This adjusted metric accounts for the influence of the number of explanatory variables in the model and provides a more realistic reflection of its generalization ability. Collectively, these metrics confirm that the proposed algorithm achieves outstanding accuracy and robustness in leaf area prediction tasks, supporting its practical value in plant phenotyping and quantitative analysis. Furthermore, a linear regression analysis was conducted using the least squares method to obtain the optimal fitting line by minimizing the sum of squared differences between the predicted and actual values. The resulting regression equation is expressed as Equation 14, where the slope a=1.0078 (Equation 15) and the intercept b=−6.6564. The final fitted equation, presented in Equation 16, quantitatively describes the relationship between manual and algorithmic measurements. As observed in **Figure 9A**, the data points are symmetrically distributed around the fitted line, suggesting a good match. **Figure 9B** illustrates a three-dimensional spatial distribution of the manual and algorithmic results, providing a visual representation of the consistency between the two measurement approaches. **Figure 9C** displays the residual plot, showing the deviations of the predicted values from the observed ones. The residuals are distributed evenly on both sides of the zero line without evident patterns, indicating the absence of systematic error and further confirming the goodness of fit. Lastly, **Figure 9D** shows that the residuals follow a normal distribution, indicating that the prediction errors are both random and unbiased, thereby affirming the robustness and reliability of the model’s predictions.

In conclusion, the YOLOv11-HSECal model demonstrates high accuracy in estimating okra leaf area, effectively supporting the practical application demands of okra seedling leaf monitoring tasks.

### 3.6 Analysis of the vigor of okra seeds and the growth status of okra seedlings under salt stress

**Figure 10:**
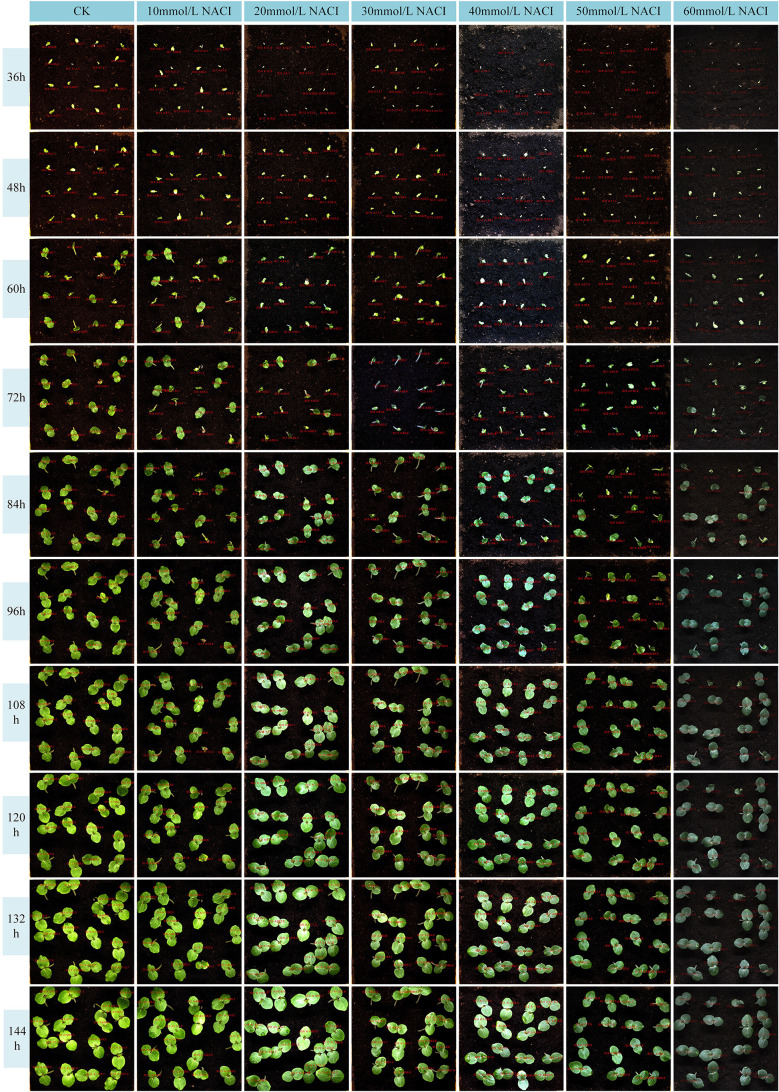
Okra seedling leaf area under different concentration of NACI solutions.

**Figure 11:**
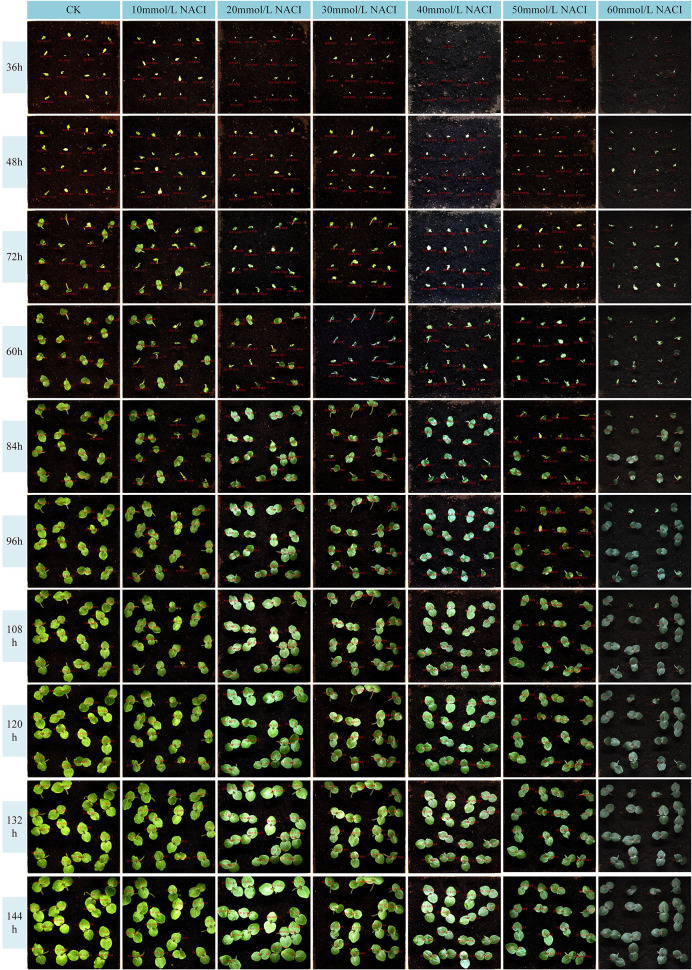
Okra seedling leaf growth rate under different concentration of NACI solutions.

**Figure 12:**
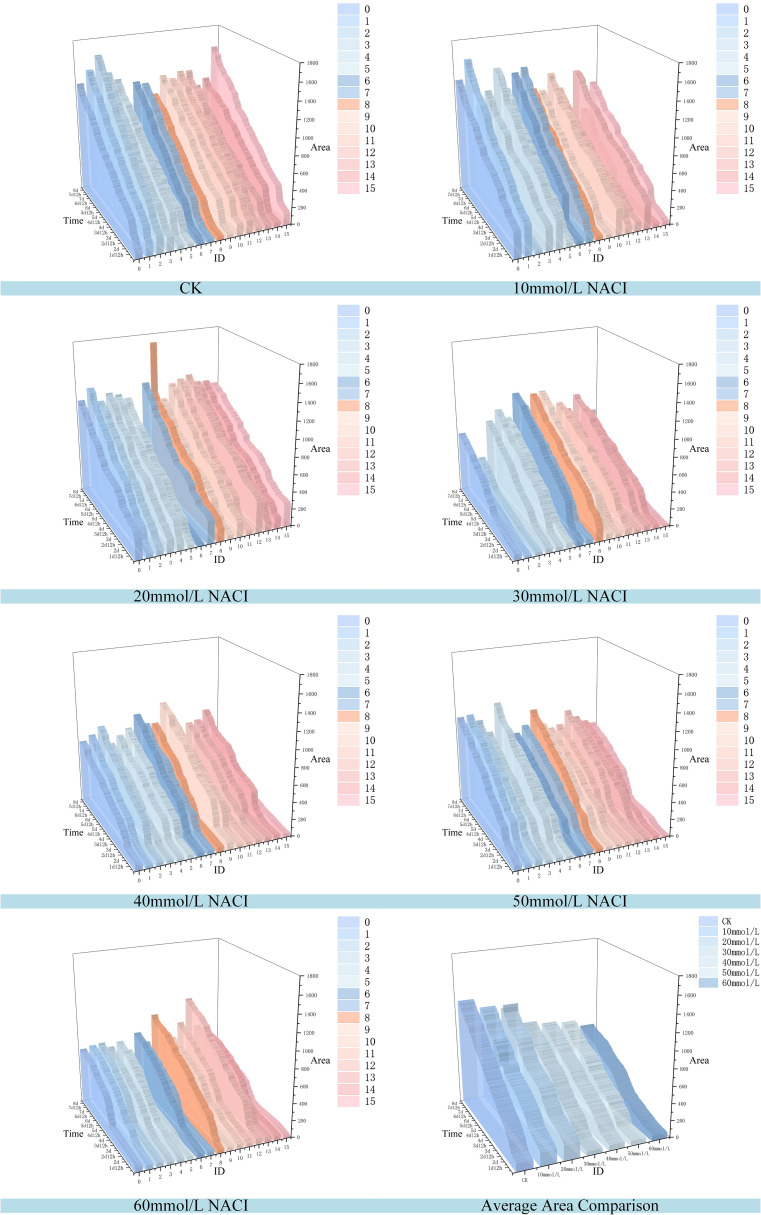
Changes of leaf area of each leaf and average leaf area of 16 leaves in the seedling stage of the culture box under different concentration of NACI solutions over time.

**Figure 13:**
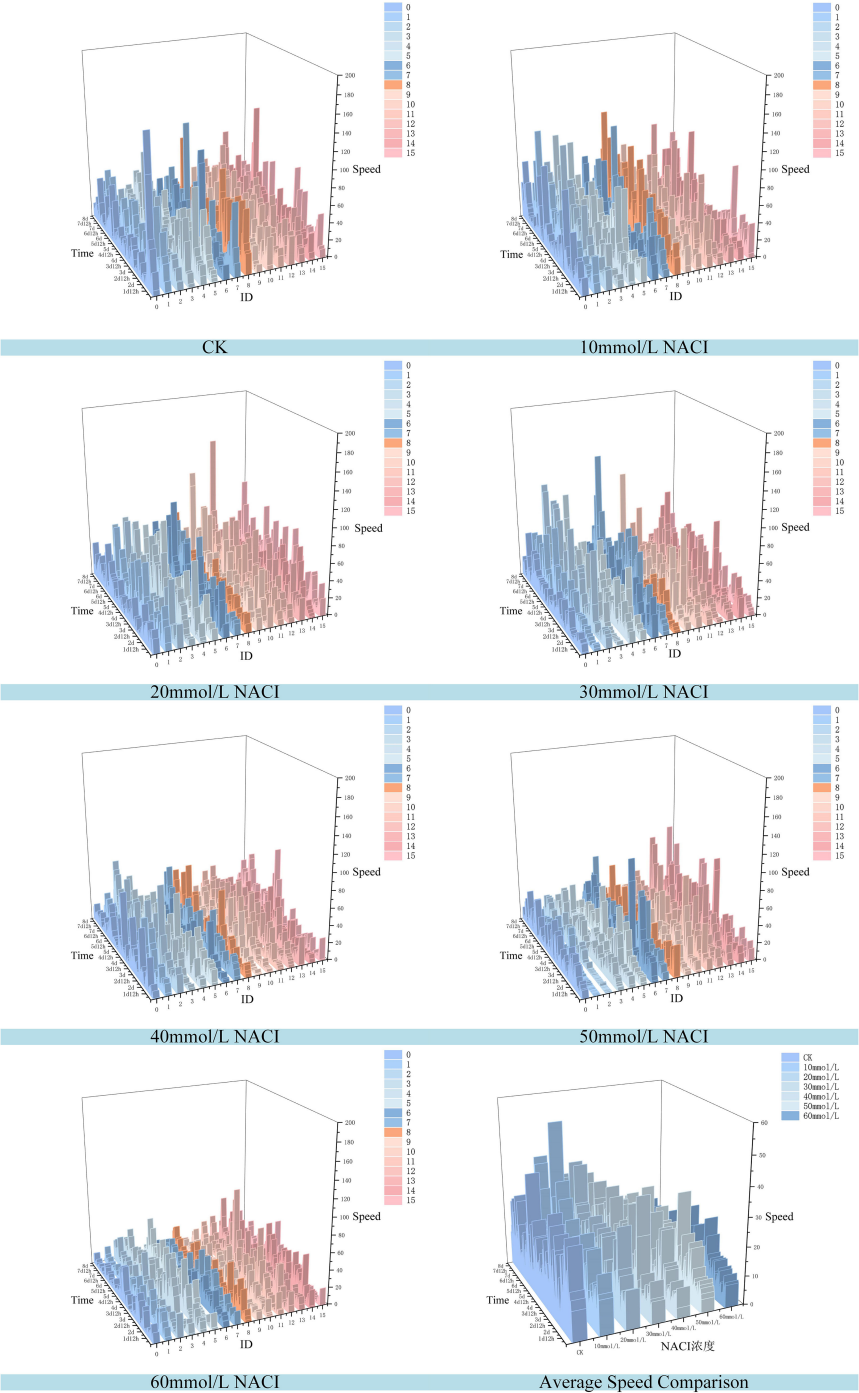
Changes of leaf growth rate of each leaf and average leaf growth rate of 16 leaves in the seedling stage of the culture box under different concentration of NACI solutions over time.

Salinity is one of the major environmental factors adversely affecting plant growth and is known to significantly reduce crop yields. In this study, we conducted experiments at the seedling stage of okra under varying concentrations of NaCl solutions. A control group was established using deionized water, while treatment groups were irrigated with NaCl solutions at concentrations of 10 mmol/L, 20 mmol/L, 30 mmol/L, 40 mmol/L, 50 mmol/L, and 60 mmol/L. Growth images were collected at ten time points, and leaf area as well as leaf growth rate were calculated. The results are illustrated in **Figures 10**, **11**. Due to inherent limitations in model precision, a very small number of negative values appeared in the calculated growth rates, which can be considered negligible in the overall analysis. **Figures 12**, **13** further illustrate the leaf growth patterns of okra seedlings under varying NaCl concentrations. As the concentration of NaCl increased, a clear downward trend was observed in both leaf area and leaf growth rate at the same developmental stage, indicating that salt stress progressively inhibited leaf expansion and growth vigor. To highlight the differences more clearly, image data from the third day of the seedling stage were analyzed. The average leaf area and real-time growth rate in the CK group were 325.175 mm² and 13.32 mm²/h, respectively. Under increasing NaCl concentrations, the average leaf area and average real-time growth rate were reduced to 241.79 mm², 138.75 mm², 87.66 mm², 75.28 mm², 66.37 mm², and 64.54 mm², and to 6.78 mm²/h, 5.63 mm²/h, 3.13 mm²/h, 2.73 mm²/h, 1.69 mm²/h, and 2.06 mm²/h, respectively.

**Figures 12**, **13** illustrate the leaf area and growth rate of okra seedlings under control conditions (CK) and at various concentrations of NaCl solution. **Figure 12** presents the dynamic changes in the area of each individual leaf and the average area of 16 leaves during the seedling stage, across different treatment groups. The monitoring period spanned from 1 day and 12 hours to 8 days (a total of 156 hours), with images captured at 15-minute intervals, resulting in 624 images per group—thereby enabling continuous full-time monitoring of okra leaf area. As shown in **Figure 12**, although a few individual leaves at each concentration deviated from the overall trend, the growth trajectories of the majority of the 16 leaves at each NaCl concentration remained consistent. With increasing NaCl concentration, the slopes of the growth curves (represented by wall plots) gradually decreased, indicating a reduction in growth vigor. Based on the data from **Figure 12**, the average leaf areas of the 16 leaves at each concentration on the second day were 268.16 mm² (CK), 231.54 mm² (10 mmol/L), 132.47 mm² (20 mmol/L), 77.1 mm² (30 mmol/L), 69.34 mm² (40 mmol/L), 64.24 mm² (50 mmol/L), and 57.28 mm² (60 mmol/L), respectively. On the fourth day, the average areas increased to 574.8 mm², 553.1 mm², 530.57 mm², 384.79 mm², 373.38 mm², 337.03 mm², and 250.11 mm², respectively. By the seventh day, the values reached 1064.57 mm², 897.75 mm², 908.53 mm², 741.17 mm², 703 mm², 657.7 mm², and 626.62 mm², respectively. These results demonstrate that, across all treatments, the average leaf area of okra seedlings increased over time. However, higher NaCl concentrations consistently resulted in smaller leaf areas when compared to lower concentrations or the CK group at the same time points, indicating that increased salinity negatively affected the growth vigor of okra seedlings.

**Figure 13** illustrates the variation in growth rate for each individual leaf and the average growth rate of 16 leaves at the okra seedling stage under CK and various NaCl solution concentrations. The observation period was consistent with that of **Figure 13**, spanning from 1 day and 12 hours to 8 days (totaling 156 hours), with images captured at 15-minute intervals, resulting in 624 images per treatment group. From the temporal trends in leaf growth rate under the seven NaCl concentrations shown in **Figure 13**, it is evident that although certain leaves exhibited phases of rapid growth within a specific period, the overall growth rates tended to stabilize over time. The early-stage growth rates were slightly lower than those in the mid-to-late stages. Leaves within the same concentration group exhibited generally consistent growth patterns. However, with increasing NaCl concentration, the height of the wall plots progressively decreased, indicating a gradual decline in both growth rate and physiological vigor. Furthermore, **Figure 13** shows that on the second day, the average growth rates of the 16 leaves under CK and NaCl treatments at 10, 20, 30, 40, 50, and 60 mmol/L were 14.22 mm²/h, 9.86 mm²/h, 9.07 mm²/h, 8.83 mm²/h, 7.05 mm²/h, 4.99 mm²/h, and 6.73 mm²/h, respectively. On the fourth day, the rates increased to 17.13 mm²/h, 18.17 mm²/h, 15.44 mm²/h, 10.3 mm²/h, 9.49 mm²/h, 7.79 mm²/h, and 9.04 mm²/h. By the seventh day, the corresponding growth rates were 25.18 mm²/h, 9.07 mm²/h, 13.94 mm²/h, 13.35 mm²/h, 1.17 mm²/h, 5.63 mm²/h, and 1.09 mm²/h. These findings suggest that okra seedlings exhibit relatively slow leaf growth during the early stages, with a noticeable increase in growth rate during the middle and late stages. The overall trend of leaf growth rate mirrors that of leaf area: at a given concentration, the growth rate is typically higher in the later stages than in the early phase. Although occasional anomalies were observed where high-concentration treatments yielded slightly higher rates than lower concentrations, the overall average growth rate consistently declined with increasing NaCl concentration. This confirms that the salt stress environment simulated by NaCl significantly inhibits both the growth capacity and developmental potential of okra seedlings.

## 4 Discussion

In this study, a full-time sequence evaluation method for assessing okra seedling vigor was developed, offering a valuable tool and reference for understanding leaf development during the seedling stage, optimizing seed treatment strategies, and supporting rapid breeding as well as precision growth management. Despite its effectiveness, the current system has several limitations. First, the light intensity within our full-time monitoring system for crop germination vigor is not yet adjustable, which restricts experimental flexibility under varying illumination conditions. To address this issue, we plan to incorporate an adjustable lighting module to facilitate data collection under different light intensities. Second, the system lacks an automatic irrigation function and currently relies on daily manual watering during the experiment. This increases the labor burden and poses challenges for maintaining consistent environmental conditions. To enhance system automation and environmental control, we intend to integrate an automatic irrigation module in future iterations. Finally, the current assessment primarily relies on leaf area and leaf growth rate as indicators of okra seedling vigor. While these metrics are valid, future work could explore the integration of additional morphological indicators, such as stem length and stem growth rate, derived from 3D stereoscopic imaging. This would enable a more comprehensive and multidimensional evaluation of seedling vigor.

## 5 Conclusion

To address the issues of large errors and low efficiency associated with traditional manual leaf area measurements, as well as the limitations of existing instance segmentation models—such as complex architecture, high parameter count, and poor robustness—this study aimed to achieve high-throughput, lightweight, and full-time monitoring of okra seedling vigor. To this end, the following research was conducted to explore a full-time seedling vigor evaluation approach for okra based on the YOLOv11-HSECal model:

1. We developed a full-time sequence crop germination vigor monitoring system capable of supporting automated and continuous monitoring of okra seedlings, encompassing dynamic data acquisition from seed germination through to seedling development. The system not only provides a stable environment with controlled light and temperature but also ensures data reliability and validity through high-throughput image acquisition and precise growth tracking. This establishes a robust foundation for assessing plant growth status. Utilizing this system, we successfully conducted a 9-day okra seedling experiment under salt stress conditions, comprising a control group (CK) and six different concentrations of NaCl solution. A total of 3,456 seedling images were collected. Following data annotation and augmentation, we constructed an image dataset capturing the growth dynamics of okra seedlings.
2. To address the task of leaf segmentation and growth evaluation, this study optimized the YOLOv11-seg model and proposed the YOLOv11-HSECal model. By integrating the HGNetv2 backbone network, the Slim-Neck feature fusion module, the EMAttention attention mechanism, and a combination of the Merge and Cal modules, the model significantly enhances segmentation accuracy, particularly for small targets and complex leaf edges, making it directly applicable for okra seedling vigor monitoring. The optimized YOLOv11-HSECal model achieves a mAP50 of 86.9%, with the number of parameters and FLOPs reduced to 2.4M and 9.3G, respectively. This not only ensures high segmentation accuracy but also substantially improves computational efficiency, thereby meeting the requirements for lightweight, high-throughput, and high-precision monitoring in agricultural applications.
3. This study innovatively introduced the leaf growth rate as a key indicator for evaluating the growth vitality of okra seedlings. By integrating both leaf area and leaf growth rate, we assessed the effects of CK and NaCl solutions at concentrations of 10, 20, 30, 40, 50, and 60 mmol/L on seedling viability. The results demonstrated that, at each concentration, the growth rate of okra leaves in the middle and late stages was consistently higher than in the early stage. Furthermore, with increasing NaCl concentration, both leaf area and growth rate significantly declined during the same growth period, confirming the inhibitory effect of salt stress on okra seedling development. By applying the YOLOv11-HSECal model, we effectively analyzed the temporal dynamics of okra leaf growth under different levels of salt stress, providing a novel approach for plant growth assessment in adverse environments.

Although the proposed full-time-series evaluation method for okra seedling vigor demonstrates promising applications in phenotypic monitoring, several limitations remain. For instance, the current platform lacks adjustable light intensity and requires manual irrigation, which compromises the consistency of environmental control and the degree of automation. Additionally, the evaluation indices are primarily based on leaf area and growth rate, without incorporating 3D structural characteristics. Future research will aim to integrate adjustable lighting and automated irrigation modules, as well as incorporate 3D phenotypic features, to enable more precise and comprehensive assessments of seedling vigor in crops.

Conclusion: This study presents a high-throughput, non-destructive, full-time, accurate, and efficient method for assessing the vigor of okra seedlings, offering a novel approach for dynamic plant growth evaluation. Additionally, it provides a practical and effective tool for monitoring plant development under salt stress conditions, thereby advancing the application and development of intelligent agricultural technologies.

## Data Availability

The raw data supporting the conclusions of this article will be made available by the authors, without undue reservation.
